# Supplementary material for: Photoelectric responsive ionic channel for sustainable energy harvesting
Source: Nat Commun. 2023 Oct 23;14:6702. doi: 10.1038/s41467-023-42584-w (PMC10593762; doi:10.1038/s41467-023-42584-w)
Supplement: Supplementary file 1 — Supplementary Information [file 41467_2023_42584_MOESM1_ESM.pdf]

# Supplementary Information

## Photoelectric Responsive Ionic Channel for Sustainable Energy Harvesting

Qing Guo,[1] Zhuozhi Lai,[1] Xiuhui Zuo,[1] Weipeng Xian,[1] Shaochun Wu,[1] Liping Zheng,[2] Zhifeng Dai,[2] Sai Wang,*[1] Qi Sun*[1]

[1]Zhejiang Provincial Key Laboratory of Advanced Chemical Engineering Manufacture Technology, College of Chemical and Biological Engineering, Zhejiang University, Hangzhou, 310027, China

[2]Key Laboratory of Surface & Interface Science of Polymer Materials of Zhejiang Province, Department of Chemistry, Zhejiang Sci-Tech University, Hangzhou 310018, China

*Email : wangsai@zju.edu.cn (SW); sunqichs@zju.edu.cn (QS)

# Experimental Details

## ***Fabrication of free-standing dye@COF Membranes***

*COF-301.* The free-standing COF-301 membrane was synthesized *via* liquid–liquid interfacial condensation of tetraphenylmethaneamine (TAM) and 2,5-dihydroxyterephthaldeyde (DHA). The DHA (4.34 mg, 0.026 mmol) ethyl acetate and mesitylene solution (V/V = 1/3, 7 mL) was gently placed on the top of the TAM (21.7 mg, 0.057 mmol) dispersed in the acetic acid aqueous solution (1 M, 7 mL). The system was kept at 35 °C for 3 d. The free-standing COF-301 membranes were obtained after being washed sequentially with ethanol and water and then dried under vacuum for further characterization.

*Hydroxynapthol blue (HB) encapsulated COF-301 membrane ($HB_x$@COF-301).* The free-standing $HB_x$@COF-301 membranes were synthesized *via* liquid–liquid interfacial condensation. The DHA (4.34 mg, 0.026 mmol) ethyl acetate and mesitylene solution (V/V = 1/3, 7 mL) was gently placed on the top of the TAM (21.7 mg, 0.057 mmol) and HB (1.9–7.6 mg $mL^{-1}$) dispersed in the acetic acid aqueous solution (1 M, 7 mL). The system was kept at 35 °C for 3 d. The free-standing $HB_x$@COF-301 membranes were obtained after being washed sequentially with ethanol and water and then dried under vacuum for further characterization.

*HB-on-COF-301.* HB-on-COF-301 was synthesized by soaking the pre-synthesized COF-301 membrane in the HB aqueous solution (5.7 mg $mL^{-1}$) for 3 d. The resulting membrane was washed extensively with ethanol and deionized water before characterization.

## ***Fabrication of $HB_x$@COF-301/PAN***

*COF-301/PAN.* COF active layers were formed *via* liquid–liquid interfacial condensation on the surface of an asymmetric polyacrylonitrile (PAN) ultrafiltration membrane. The PAN support was vertically placed in the middle of a homemade diffusion cell, resulting in each volume of 7 $cm^3$. An acetic acid aqueous solution (1 M, 7 mL) of TAM (21.7 mg, 0.057 mmol), and the ethyl acetate and mesitylene solution (V/V = 1/3, 7 mL) of DHA (4.34 mg, 0.026 mmol) were separately introduced into the two sides of the diffusion cell. The reaction mixture was kept at 35 °C for 3 d. The resulting membrane was rinsed with methanol and ethanol to remove any residual monomers and the catalyst. Finally, each membrane was rinsed with dichloromethane for 24 h and then used for assembling with PDMS elastomer or air-dried for physicochemical characterization.

*$HB_{1.9}$@COF-301/PAN.* The PAN support was vertically placed in the middle of a homemade diffusion cell, resulting in each volume of 7 $cm^3$. An acetic acid aqueous solution (1 M, 7 mL) of TAM (21.7 mg, 0.057 mmol) and HB (13.3 mg), and the ethyl acetate and mesitylene solution (V/V = 1/3, 7 mL) of DHA (4.34 mg, 0.026 mmol) were separately introduced into the two sides of the diffusion cell. The reaction mixture was kept at 35 °C for 3 d. The resulting membrane was rinsed with methanol and ethanol to remove any residual monomers and the catalyst. Finally, each membrane was rinsed with dichloromethane for 24 h and then used for assembling with PDMS elastomer or air-dried for physicochemical characterization.

*$HB_{3.8}$@COF-301/PAN.* The PAN support was vertically placed in the middle of a homemade diffusion cell, resulting in each volume of 7 $cm^3$. An acetic acid aqueous solution (1 M, 7 mL) of TAM (21.7 mg, 0.057 mmol) and HB (26.6 mg), and the ethyl acetate and mesitylene solution (V/V = 1/3, 7 mL) of DHA (4.34 mg, 0.026 mmol) were separately introduced into the two sides of the diffusion cell. The reaction mixture was kept at 35 °C for 3 d. The resulting membrane was rinsed with methanol and ethanol to remove any residual monomers and the catalyst. Finally, each membrane was rinsed with dichloromethane for 24 h and then used for assembling with PDMS elastomer or air-dried for physicochemical characterization.

*$HB_{7.6}$@COF-301/PAN.* The PAN support was vertically placed in the middle of a homemade diffusion cell, resulting in each volume of 7 $cm^3$. An acetic acid aqueous solution (1 M, 7 mL) of TAM (21.7 mg, 0.057 mmol) and HB (53.2 mg), and the ethyl acetate and mesitylene solution (V/V = 1/3, 7 mL) of DHA (4.34 mg, 0.026 mmol) were separately introduced into the two sides of the diffusion cell. The reaction mixture was kept at 35 °C for 3 days. The resulting membrane was rinsed with methanol and ethanol to remove any residual monomers and the catalyst. Finally, each membrane was rinsed with dichloromethane for 24 h and then used for assembling with PDMS elastomer or air-dried for physicochemical characterization.

## ***Supplementary Data Items***

**Supplementary Table 1 | Representative energy conversion systems based upon selective ion transport.**

| Materials | System | Power density (W m$^{-2}$) | Resistance (kΩ) | External stimuli |
|---|---|---|---|---|
| **Osmotic energy** | | | | |
| COF−$SO_3$H−24 and COF−QA−24 membrane[1] | 0.5 M/0.01 M NaCl | 43.2 | 2.5 | |
| TpEB@TpPa-$SO_3$Na-300 membrane[2] | 0.5 M/0.01 M NaCl | 19.2 | 5.5 | |
| $Ti_3C_2T_x$ MXene ionic diode membranes[3] | 0.5 M/0.01 M NaCl | 8.6 | 10 | |
| 2DPI membrane[4] | 0.5 M/0.01 M NaCl | 65.2 | 2000 | |
| CMWs/AAO membrane[5] | 0.5 M/0.01 M NaCl | 2.78 | 10 | |
| C2DP-Por [6] | 0.5 M/0.01 M KCl | 3.6 | | |
| Poly(ether sulfone) bipolar membrane[7] | 0.5 M/0.01 M NaCl | 6.2 | 10 | |
| Nanocomposite membranes (BN)[8] | 0.5 M/0.01 M NaCl | 5.9 | | |
| Black phosphorus membranes[9] | 0.6 M/0.004 M NaCl | 4.7 | 40 | |
| Single-pore $MoS_2$[10] | 1 M/1 mM KCl | 1000000 | 9400 | |
| ZnTPP-COF[11] | 0.5 M/0.01 M NaCl | 135.8 | 5000 | |
| $JG_{80.1}$@COF/PAN[12] | 0.5 M/0.01 M NaCl | 51.4 | 14 | |
| **Chemical energy and osmotic energy** | | | | |
| Titanium Carbides Membrane[13] | 0.5/0.01 M NaCl (1 M HCl/KOH) | 0.53 (7.89) | 10 (1) | |
| **Thermo-osmotic energy** | | | | |
| RED-MED system[14] | 4.5 M/0.05 M NaCl | 4 (14) | 132 | 80 K |
| $Ti_3C_2T_x$ MXene/BN (MXBN) membrane[15] | 0.5 M/0.01 M NaCl | 0.8 (6.2) | 60 | 60 K |
| GO/Silk nanofiber membrane[16] | 0.5 M/0.01 M NaCl | 5.07 (9) | 35 | 40 K |
| AAO nanochannels[17] | 0.1 mM/0.1 mM KCl[a] | 25.5 | 50 | 30 K |
| Solar thermoelectric nanofluidic[18] | | ($4.5\times10^{-5}$) | | 35 K |
| Ultrasmall silica nanochannels[19] | 0.5 M/0.01 M NaCl | 1.0 (1.404) | 20 | 10 K |
| Montmoril-modification lamellae[20] | 1 M/1 mM KCl | 0.15 (0.34) | 10 | 30 K |
| PCTE membrane[21] | 10 mM/1 mM KCl | 74.8 nW (177.5) | | 25 K |
| PET membrane[22] | 1 M/1 mM KCl | (56.22% enhanced) | | 30 K |
| SPEEK/PES blend membrane[23] | 0.5 M/0.01 M LiBr | 9.26 (16.50) | 30 (7) | 30 K |
| COF-$(SO_3Na)_1$/PAN[24] | 0.5 M/0.01 M NaCl | 97 (231) | 8.2 (5.6) | 60 K |
| **Light energy, chemical energy, and osmotic energy** | | | | |
| MXene/CNF composite membrane[25] | 0.5/0.01 M NaCl and 0.5 M HCl/0.01 M NaOH | 76.09 (87.23) | 4 (3) | 260 mW cm$^{-2}$ |

| | | | | |
|---|---|---|---|---|
| **Light energy** | | | | |
| artificial liquid membrane[26] | 0.1 M HCl | 6.5 (8.5) | | 10 mW cm$^{-2}$ |
| carbon nitride nanotube membrane[27] | 0.001 M KCl | 1.2 mW m$^{-2}$ | 400 | 380 mW cm$^{-2}$ |
| **Light energy and osmotic energy** | | | | |
| Porphyrin MOF Membrane[28] | 3 M/1 mM NaCl | 6.26 (7.74) | 20 (10) | 100 mW cm$^{-2}$ |
| $Ti_3AlC_2$ MXene Membranes[29] | 0.5/0.01 M NaCl | 0.75 (1.75) | 5000 (4000) | 870 mW cm$^{-2}$ |
| $MoS_2$ Nanopores[30] | 100 mM/1 mM | 12.41 pW (28.67) | | 752 μW cm$^{-2}$ |
| porphyrin-based nanochannels[31] | 0.5/0.01 M NaCl | 2.16 (4.56) | 10 (9) | 100 mW cm$^{-2}$ |
| $TiO_2/C_3N_4$ heterojunction[32] | 0.1 M-0.001 M KCl | 21(28) μA cm$^{-2}$ | | 300 mW cm$^{-2}$ |
| **This work** | 0.5 M/0.01 M NaCl | 75.4 (129) | 1260 (435) | 120 mW cm$^{-2}$ |

The data in parentheses refer to the corresponding value in the presence of an external stimulus.

**Supplementary Table 2 | The dye content in the free-standing $HB_x$@COF-301 membranes and the corresponding charge density estimated based on the ionic sites.**

| Membrane | HB content (mmol $g^{-1}$) | Charge density (C $m^{-2}$) |
|---|---|---|
| COF-301 | -- | -- |
| $HB_{1.9}$@COF-301 | 0.181 | -0.0448 |
| $HB_{3.8}$@COF-301 | 0.296 | -0.0838 |
| $HB_{5.7}$@COF-301 | 0.324 | -0.0910 |
| $HB_{7.6}$@COF-301 | 0.337 | -0.0941 |

The surface charge density of $HB_x$@COF-301 was calculated using the following equations:

$$m_{HB} + m_{COF-3} = 1 \qquad (1)$$

$$(M_{TAM} + 2M_{DHA})n_{(COF-3\ )} = m_{COF-3} \qquad (2)$$

$$\frac{m_{HB}}{M_{HB}} = n_{HB} \qquad (3)$$

$$\xi = \frac{n_{HB}}{n_{(COF-3\ )}} \times 100\% \qquad (4)$$

where $m_{HB}$ (mg $g^{-1}$) and $m_{COF-301}$ (mg $g^{-1}$) is the content of HB and COF-301 in the membrane respectively. $M$ (g $mol^{-1}$) is the molar mass, $n$ is the amount of substance, and $\xi$ is the volume density of HB in the COF-301.

According to the function between the charge quantity ($e$ =1.6 × $10^{-19}$ C) of each HB molecule and the pore area of COF-301 ($S$, $m^2$), the surface charge density ($\Sigma$) of $HB_x$@COF-301 can be obtained using the following equation:

$$\Sigma = \frac{e\xi}{S} \qquad (5)$$

**Supplementary Table 3 | Zeta potentials of various membranes.**[a]

| Membrane | Zeta potential (mV) |
|---|---|
| PAN | -19.1 |
| COF-301/PAN | -22.6 |
| $HB_{1.9}$@COF-301/PAN | -31.7 |
| $HB_{3.8}$@COF-301/PAN | -38.4 |
| $HB_{5.7}$@COF-301/PAN | -45.1 |
| $HB_{7.6}$@COF-301/PAN | -48.9 |

[a]Measurements were carried out with 1.0 mmol $L^{-1}$ KCl aqueous solution at (25.0 ± 1.0) °C at a pH of 6.5. Data were collected for four cycles at each measuring point. Surface zeta potential was calculated according to the Helmholtz–Smoluchowski equation.

**Supplementary Table 4 | The dependence of Debye screening length on the concentration of KCl solution.**

| Concentration (mM) | 1000 | 100 | 10 | 1 | 0.1 |
|---|---|---|---|---|---|
| Debye length (nm)[a] | 0.3 | 0.96 | 3.03 | 9.59 | 30.32 |

[a]The Debye length is defined as

$$\lambda_D = \left(\frac{\varepsilon_r \varepsilon_0 k_B T}{2 N_A e^2 I}\right)^{1/2} \quad (6)$$

where $\varepsilon_r$ and $\varepsilon_0$ are the vacuum and relative permittivity, respectively, $k_B$ is the Boltzmann constant, $T$ is the absolute temperature, $e$ is the elementary charge, $N_A$ is the Avogadro number, and $I$ is the ionic strength of the solution.

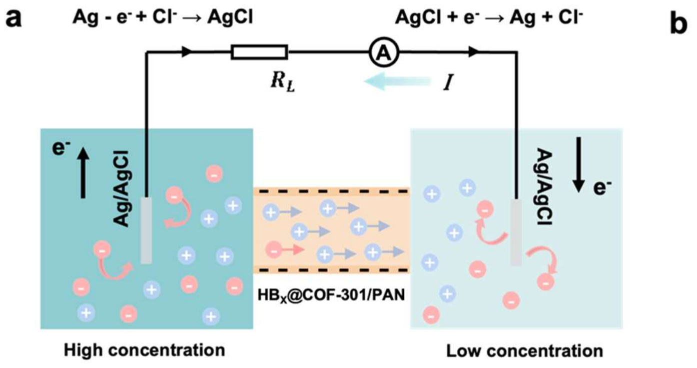


**Supplementary Figure 1| Harvesting of salinity energy via the $HB_x$@COF-301/PAN membrane.** (a) Schematic illustration of harvesting energy from salt concentration gradient via the $HB_x$@COF-301/PAN membrane. A selective membrane favors the transport of counterions from the high-concentration reservoir (left) to the low-concentration reservoir (right) and thus generates an ionic current. The negatively charged $HB_x$@COF-301/PAN membranes, which favor the flux of cations over anions, are shown. The difference between the cationic flux and anionic flux is the ionic current, $I$. The Ag/AgCl electrodes are necessary to convert the ionic current to electrical current via redox reactions, thereby closing the electrical circuit. (b) Equivalent circuit representing the harvesting of energy by an external load, $R_L$, where $R_{int}$ is the internal resistance of the nanopore-based power generator and $\Delta E_m$ is the membrane potential.

We integrated a pair of redox electrodes (Ag/AgCl) into the system to facilitate redox reactions that help balance the charge as cations selectively migrate across the membrane and accumulate in the low-salinity reservoir. This configuration ensures that anions remain in the high-salinity reservoir, thereby maintaining charge separation. This illustration visually demonstrates how the redox electrodes enable the conversion of ionic charge flux into an electrical current while maintaining charge separation and electric neutrality. The redox reactions occurring at the electrodes not only address the concern of electric neutrality but also contribute to the efficient generation of blue energy. By connecting an external load to the system, we can harness the potential energy difference between the high-salinity and low-salinity reservoirs to generate electricity effectively.

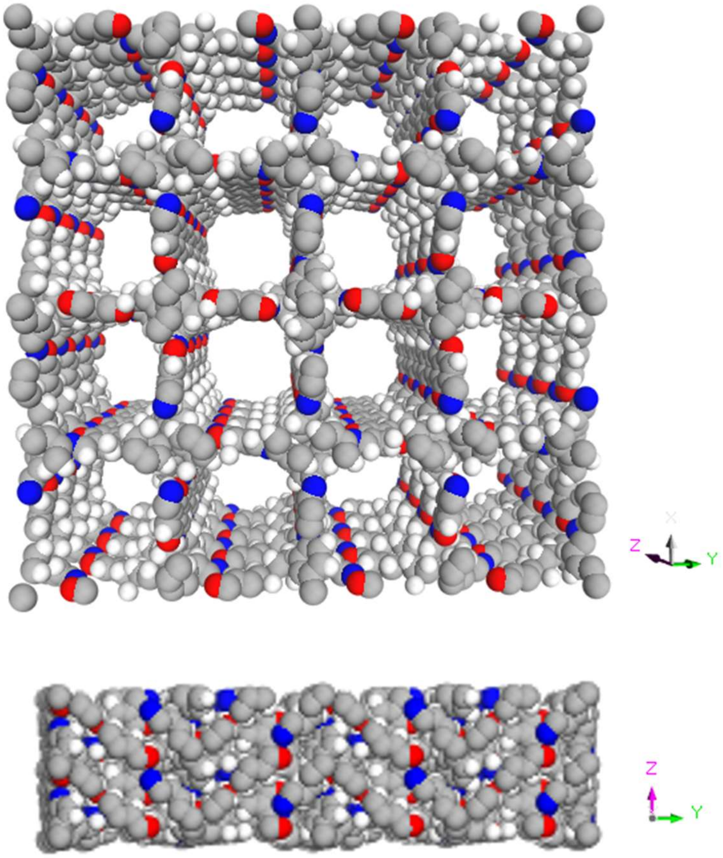


**Supplementary Figure 2 | Graphic view of COF-301 (blue, N; grey, C; red, O; white, H).**

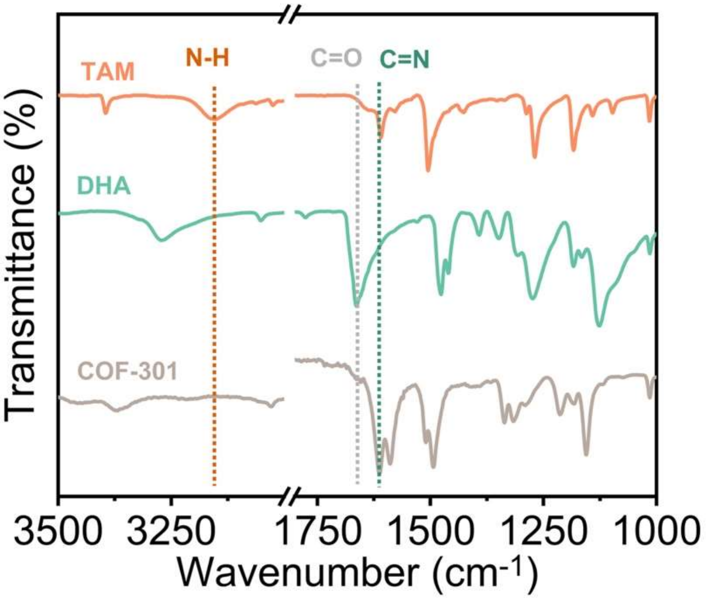


**Supplementary Figure 3 | IR spectra of the free-standing COF-301 membrane and the corresponding monomers.**

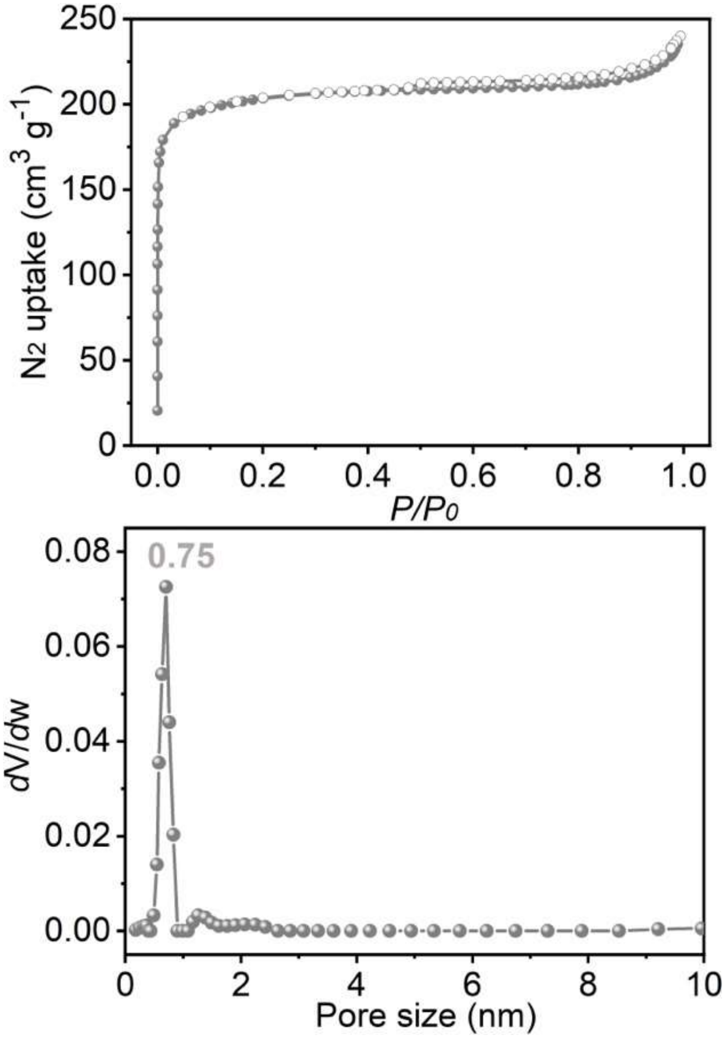


**Supplementary Figure 4 | $N_2$ sorption isotherms collected at 77 K (top) and the corresponding pore size distribution of COF-301 (bottom).** The BET surface area was calculated to be 564 $m^2$ $g^{-1}$ and the pore size distribution was derived from the nonlocal density functional theory (NLDFT).

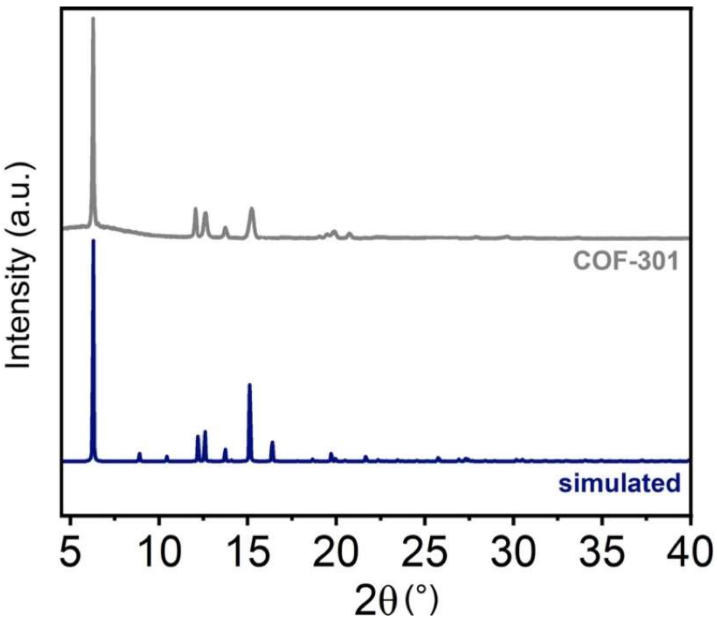


**Supplementary Figure 5 | XRD patterns of the experimental and simulated COF-301.**

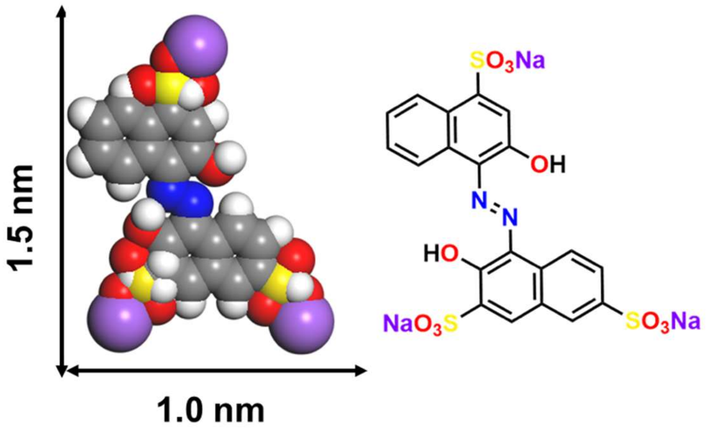


**Supplementary Figure 6 | The chemical structure and dimentions of the HB molecule (blue, N; grey, C; red, O; white, H; yellow, S; purple, Na).**

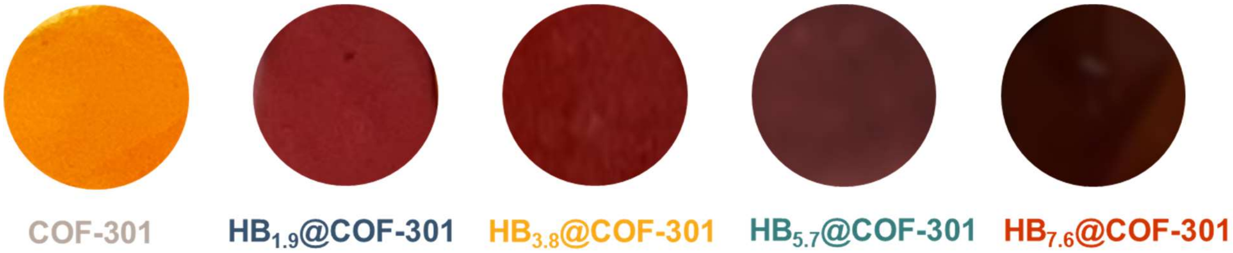


**Supplementary Figure 7 | Digital photos of the free-standing COF-301, $HB_{1.9}$@COF-301, $HB_{3.8}$@COF-301, $HB_{5.7}$@COF-301, and $HB_{7.6}$@COF-301 membranes, respectively.**

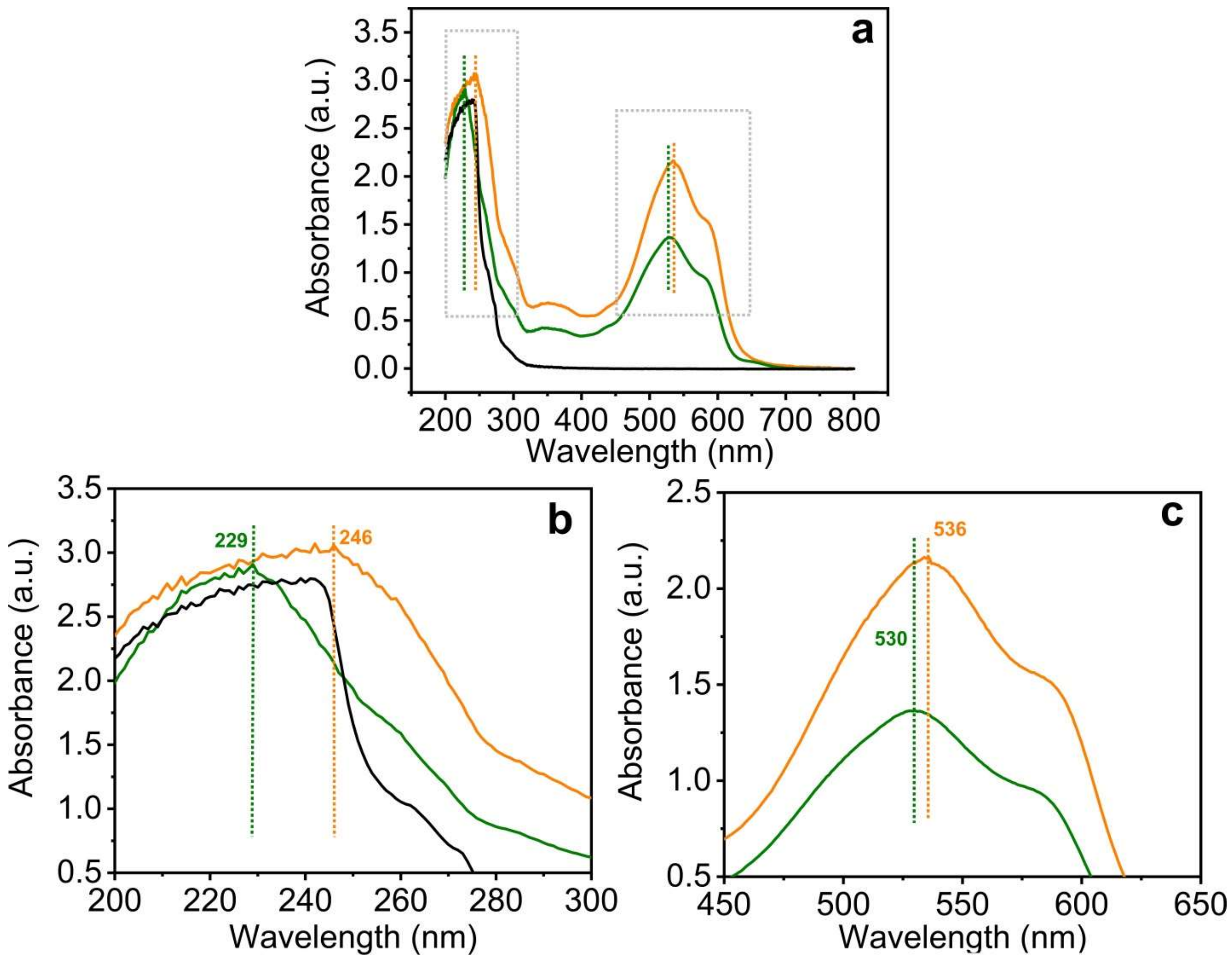


**Supplementary Figure 8 | UV-vis spectra.** (a) TAM-acetic acid-aqueous solution (black line), HB aqueous solution (olive green), and the mixture of TAM-acetic acid-aqueous solution and HB aqueous solution (orange line), (b) enlarged section (200–300 nm) of gray rectangle in (a), and (c) enlarged section (450–650 nm) of gray rectangle in (a).

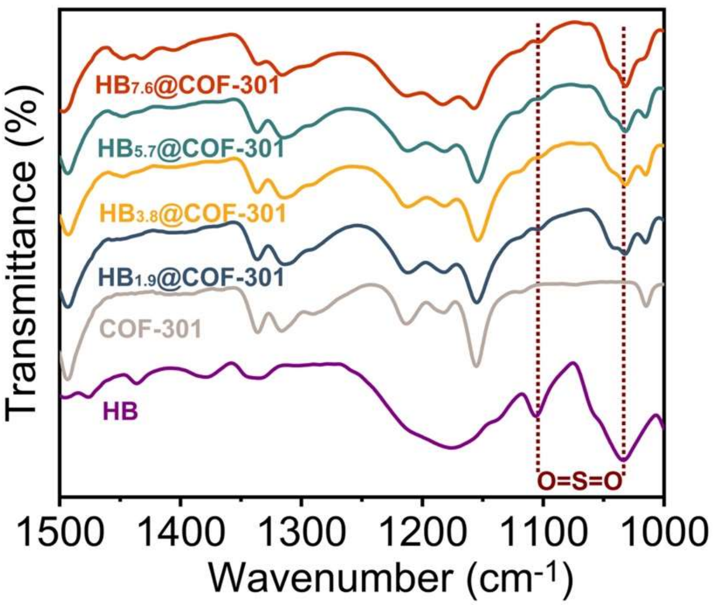


**Supplementary Figure 9 | IR spectra of the HB molecule and the free-standing COF membranes.**

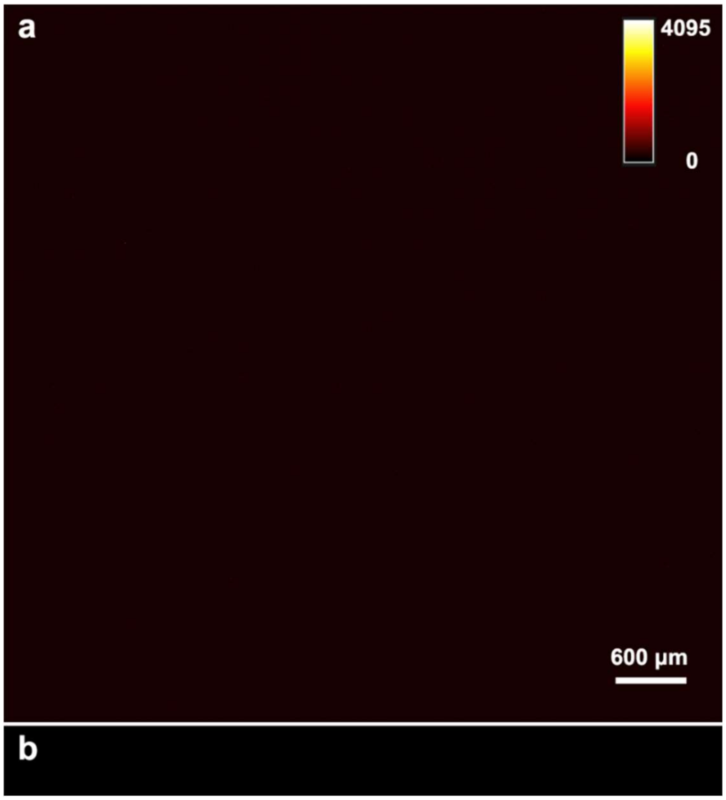


**Supplementary Figure 10 | Confocal microscopy images.** (a) Top view and (b) cross-sectional view of the free-standing COF-301 membrane. The black views suggest that there are no fluorescent molecules in the membrane. The scale bar of (b) is the same as that in (a).

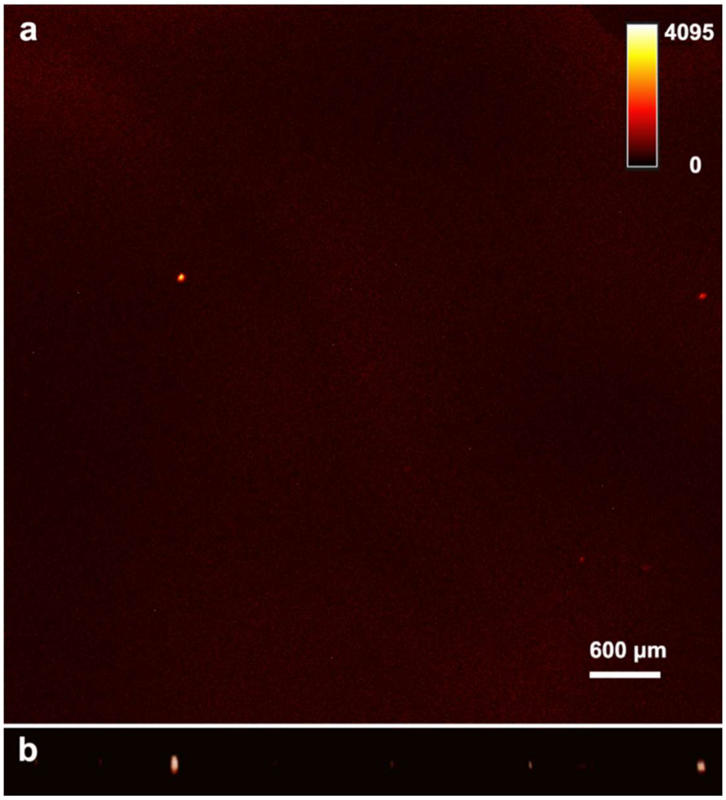


**Supplementary Figure 11 | Confocal microscopy images.** (a) Top view and (b) cross-sectional view of the HB-on-COF-301 membrane. The scale bar of (b) is the same as that in (a).

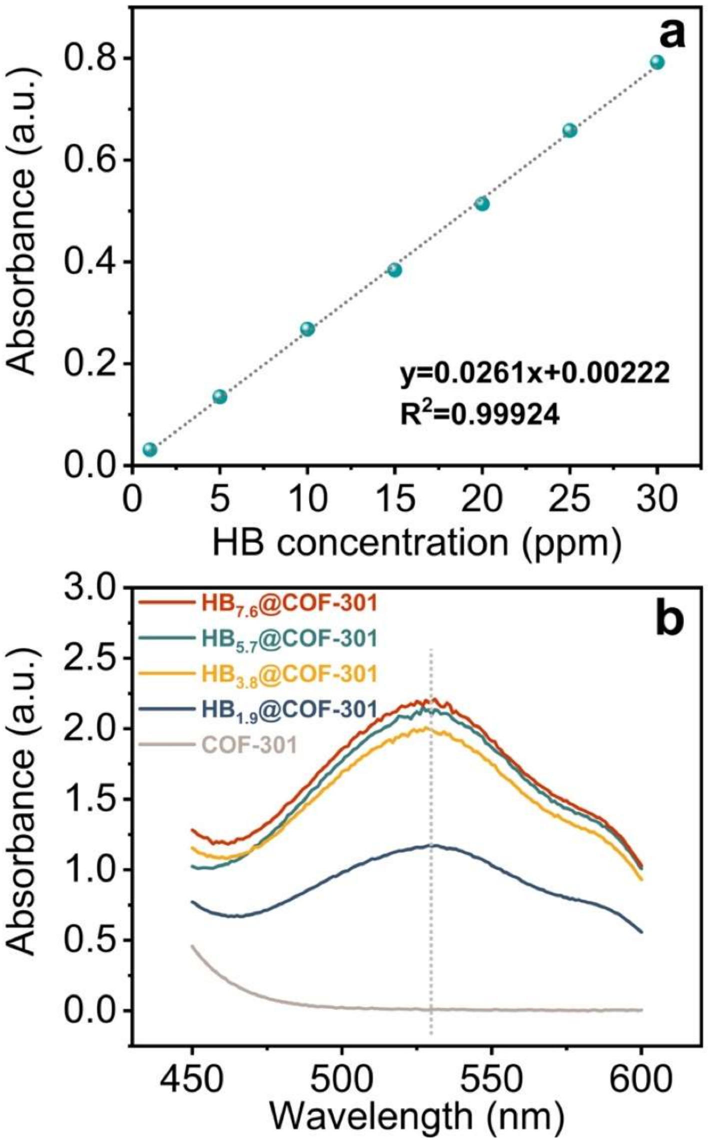


**Supplementary Figure 12 | UV-vis spectra.** (a) A representative standard curve showing the absorbance of different concentrations of HB using UV-vis spectroscopy, and (b) the HCl digested free-standing COF-301 and $HB_x$@COF-301 membranes.

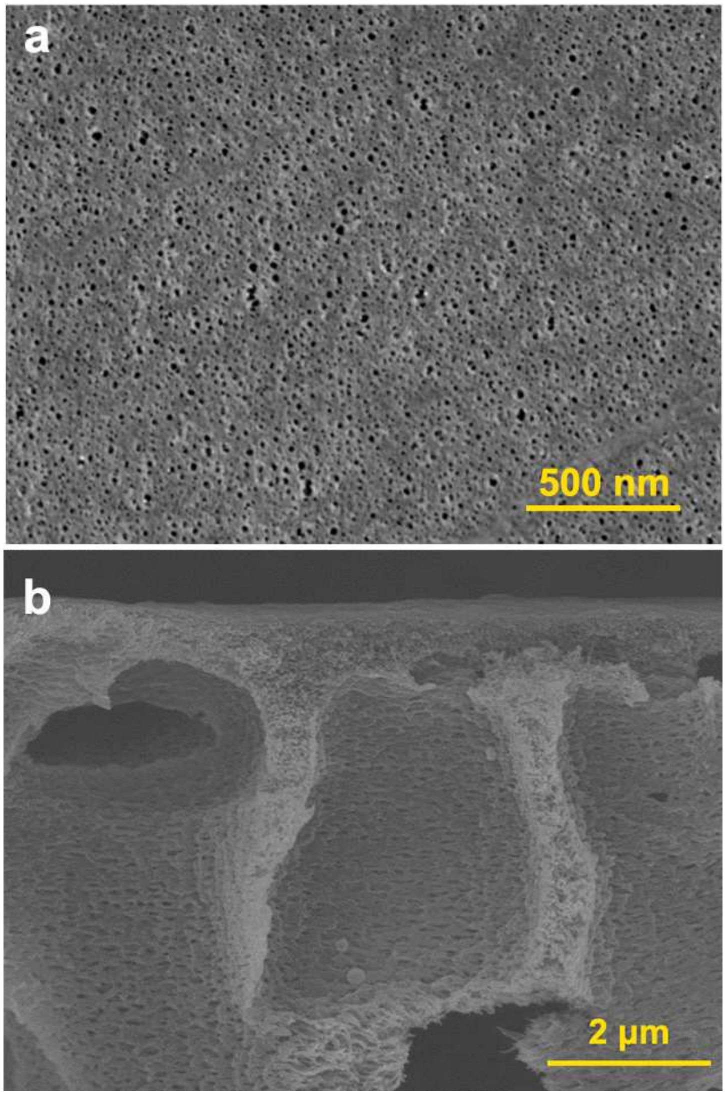


**Supplementary Figure 13 | SEM images.** (a) Top-view and (b) cross-sectional SEM images of the PAN membrane.

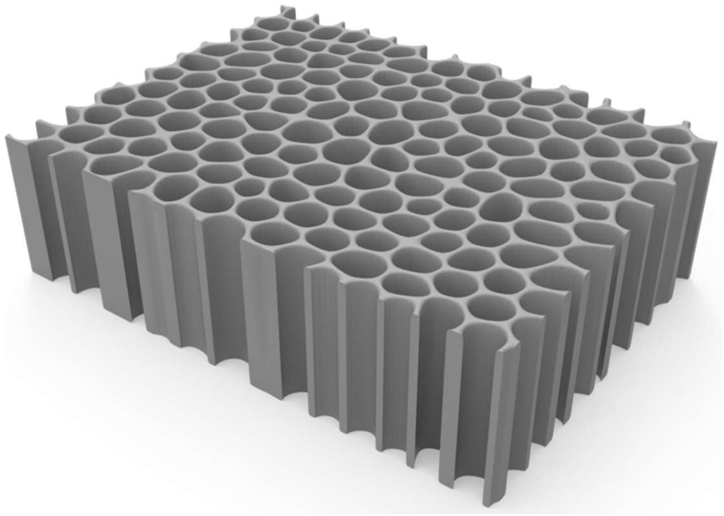

**Supplementary Figure 14 | Schemetic illustration of the pore structure of the PAN membrane.**

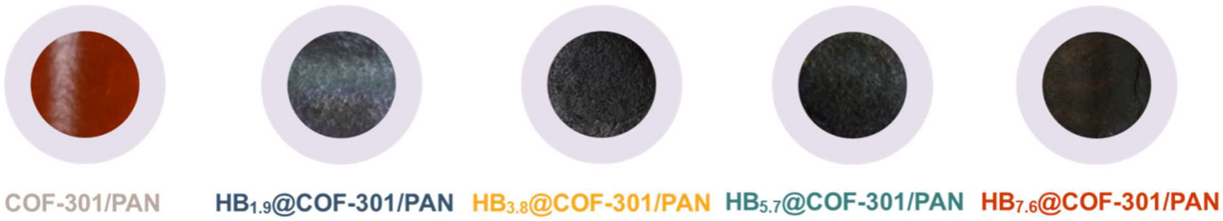


**Supplementary Figure 15 | Digital photos.** COF-301/PAN, $HB_{1.9}$@COF-301/PAN, $HB_{3.8}$@COF-301/PAN, $HB_{5.7}$@COF-301/PAN, and $HB_{7.6}$@COF-301/PAN, respectively from the left to right.

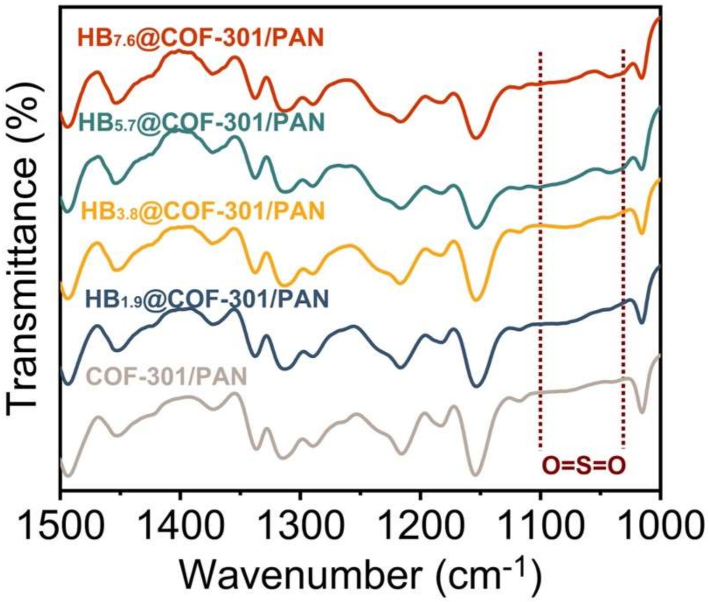


**Supplementary Figure 16 | IR spectra of the COF-301/PAN and $HB_x$@COF-301/PAN membranes.**

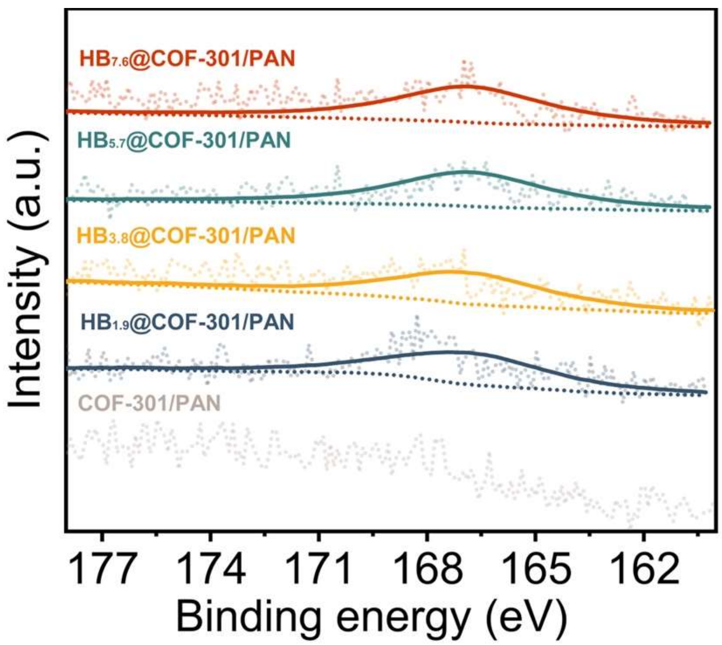


**Supplementary Figure 17 | S *2p* XPS spectra.** The calculated integral areas of S *2p* are 0, 1588, 1607, 1680, and 1760 for COF-301/PAN, $HB_{1.9}$@COF-301/PAN, $HB_{3.8}$@COF-301/PAN, $HB_{5.7}$@COF-301/PAN, and $HB_{7.6}$@COF-301/PAN, respectively.

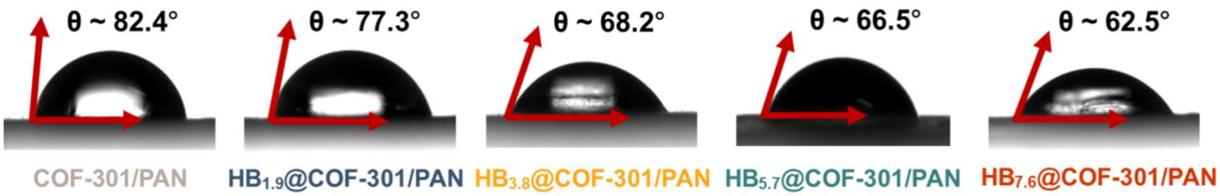


**Supplementary Figure 18 | Photographs of water droplets on the composite membranes.**

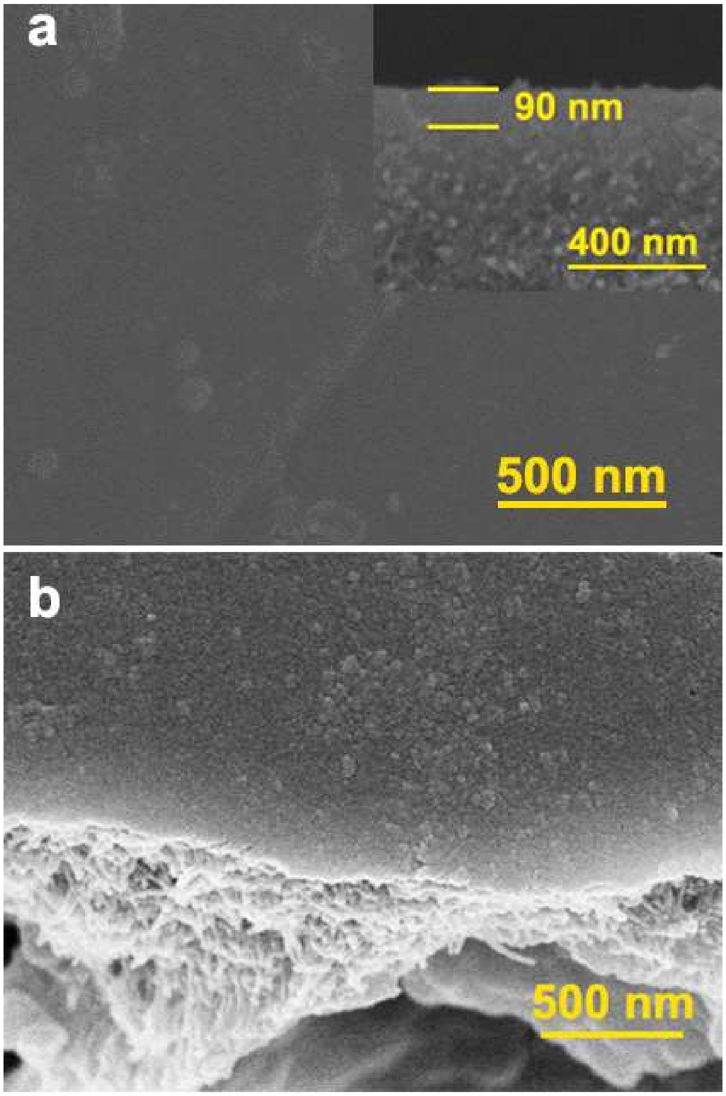


**Supplementary Figure 19 | SEM images.** (a) Top view (inset: the thickness of the membrane) and (b) cross-sectional view of COF-301/PAN.

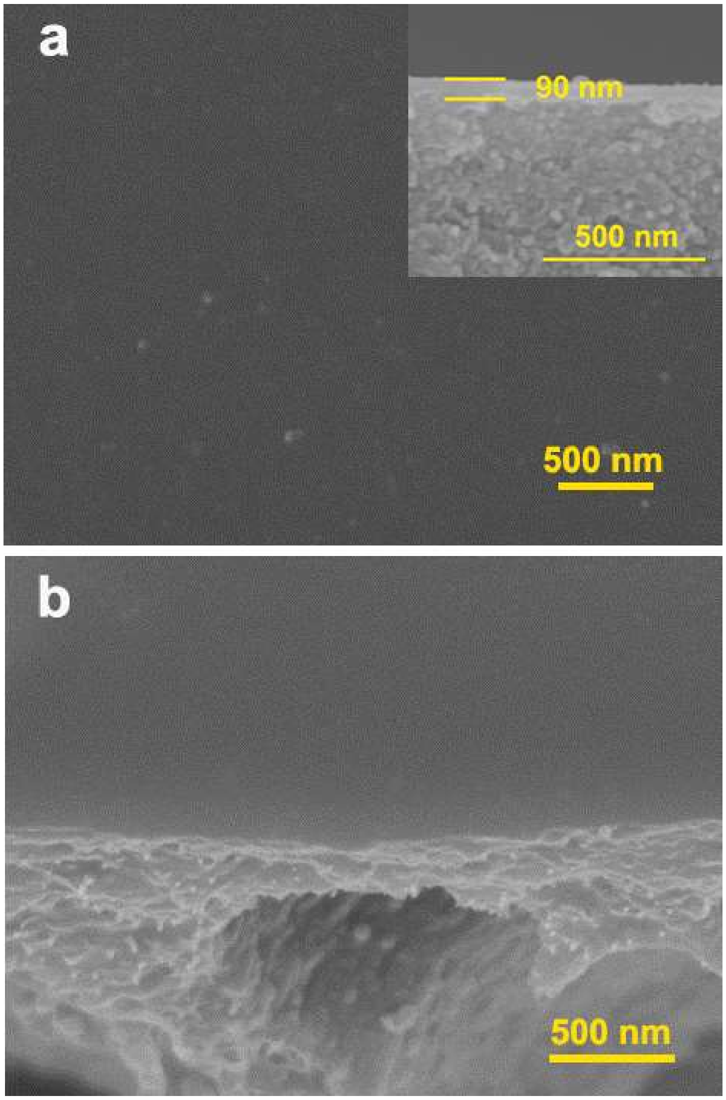


**Supplementary Figure 20 | SEM images.** (a) Top view (inset: the thickness of the membrane) and (b) cross-sectional view of $HB_{1.9}$@COF-301/PAN.

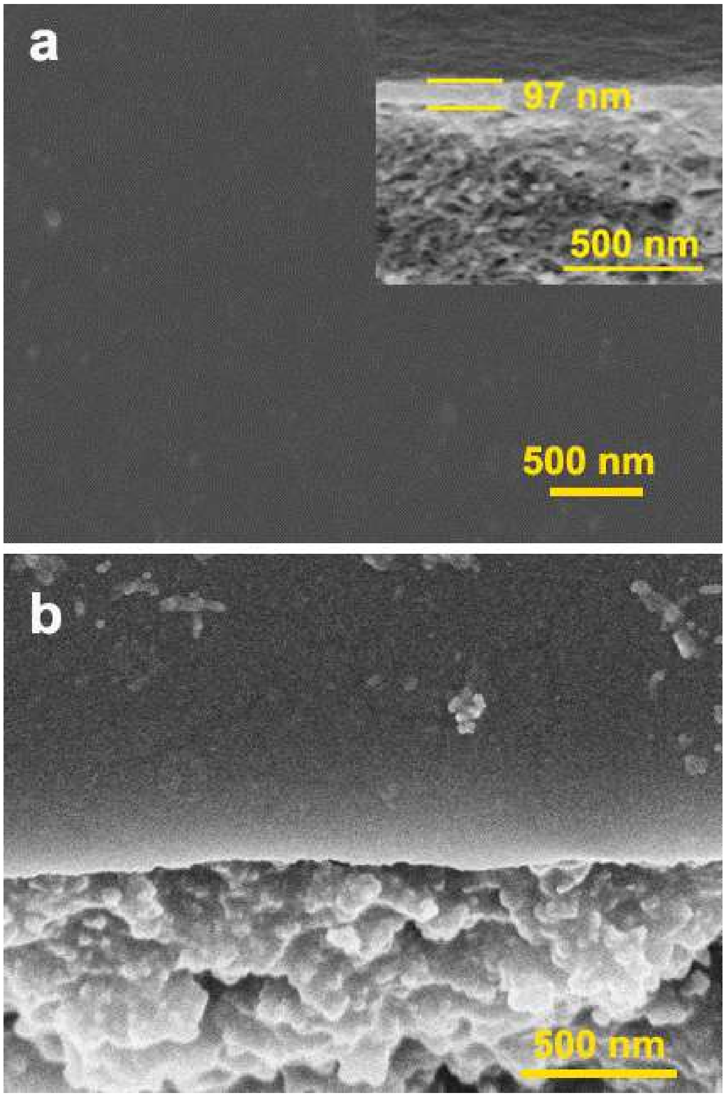


**Supplementary Figure 21 | SEM images.** (a) Top view (inset: the thickness of the membrane) and (b) cross-sectional view of $HB_{3.8}$@COF-301/PAN.

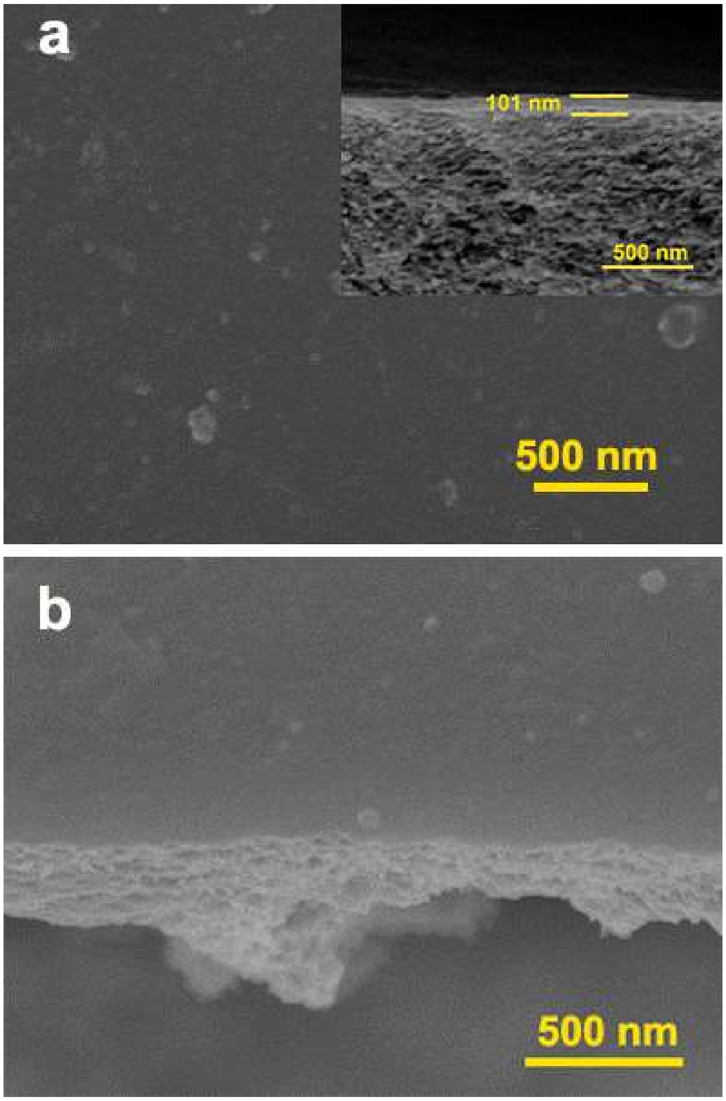


**Supplementary Figure 22 | SEM images.** (a) Top view (inset: the thickness of the membrane) and (b) cross-sectional view of $HB_{5.7}$@COF-301/PAN.

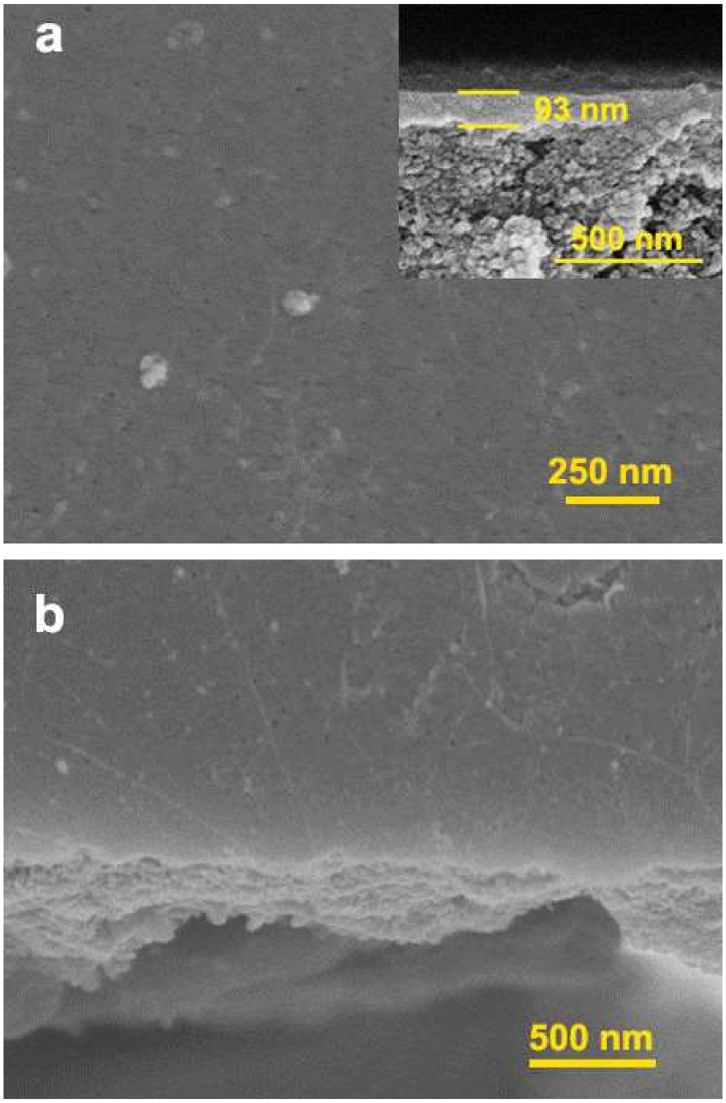


**Supplementary Figure 23 | SEM images.** (a) Top view (inset: the thickness of the membrane) and (b) cross-sectional view of $HB_{7.6}$@COF-301/PAN.

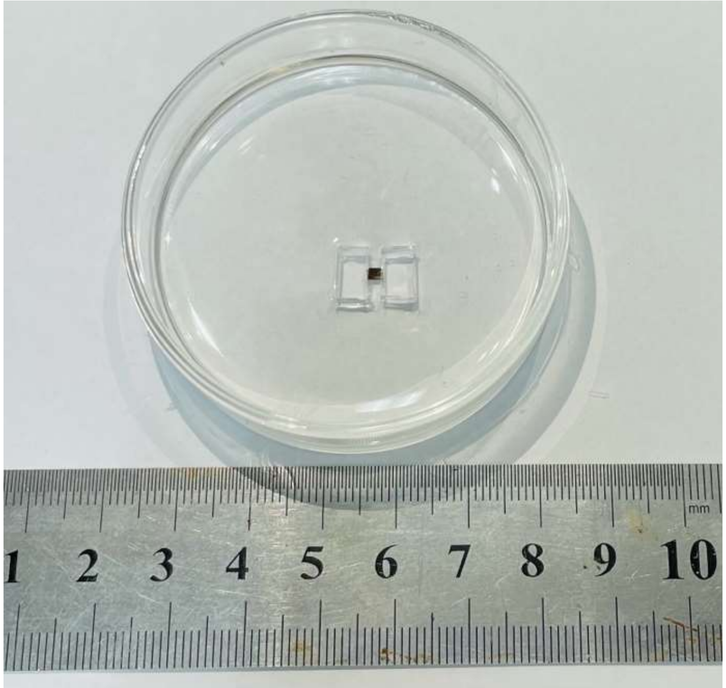


**Supplementary Figure 24 | Photograph of the fabricated nanofluidic device.**

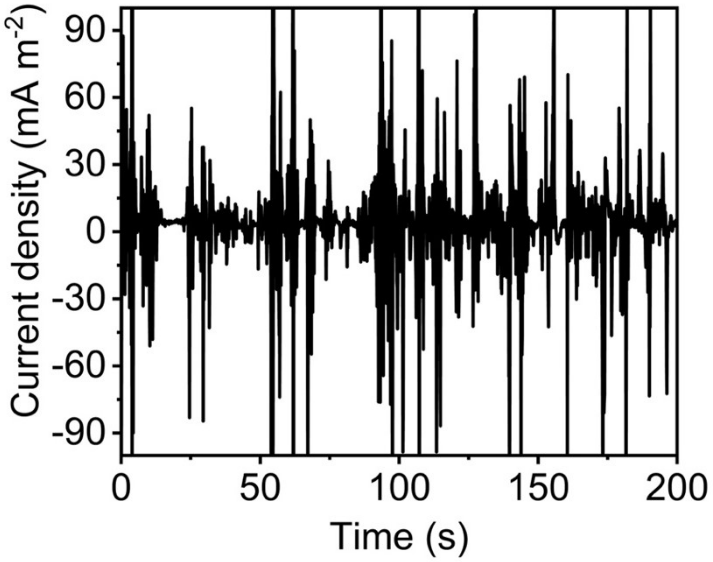


**Supplementary Figure 25 | $I$–$t$ curve of the nanofluidic device assembled by PAN recorded in symmetric KCl aqueous solution (1 mM).** No detectable current was observed for PAN, confirming the absence of percolated leakage pathways along the direction of ionic current.

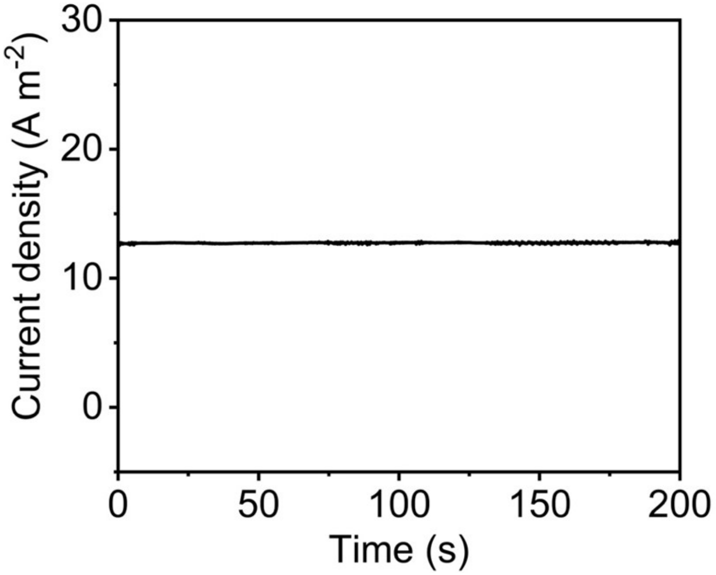


**Supplementary Figure 26 | *I–t* curve of the nanofluidic device assembled by $HB_{5.7}$@COF-301/PAN recorded in symmetric KCl aqueous solution (1 mM).** The detected stable current suggests that the ion can pass through the membrane.

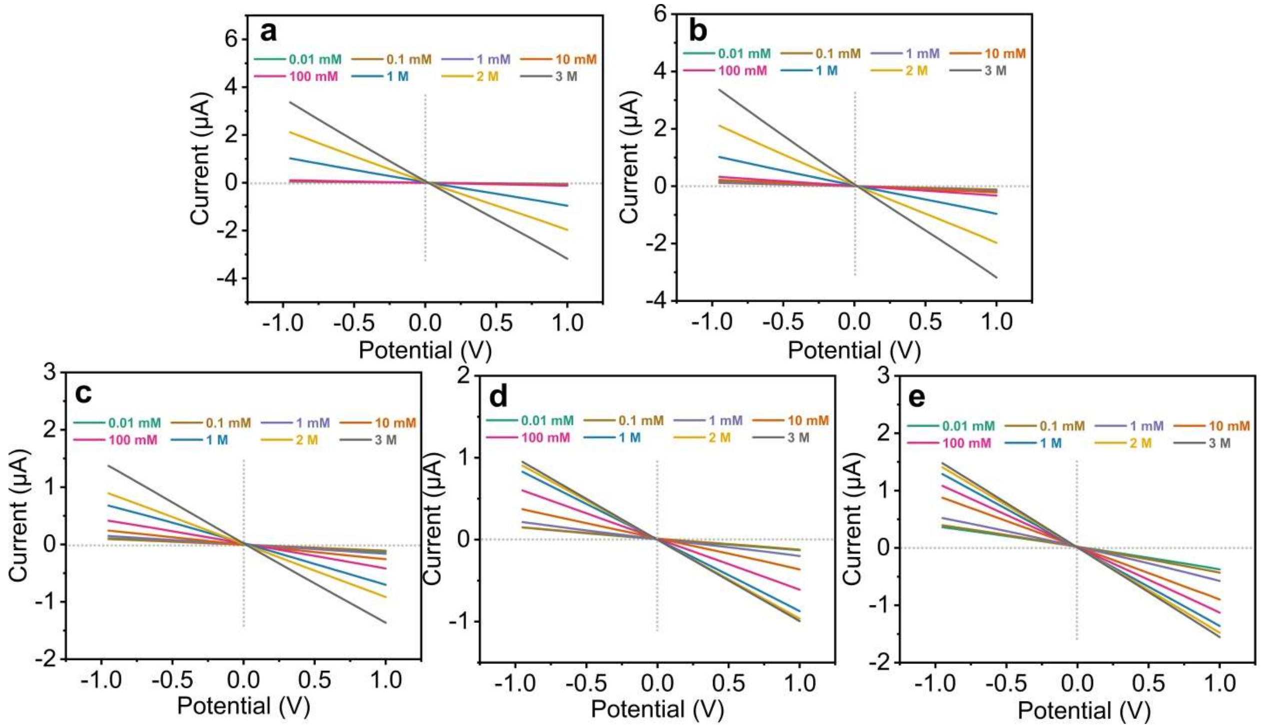


**Supplementary Figure 27 | $I$–$V$ curves of the nanofluidic devices assembled by $HB_x$@COF-301/PAN recorded in KCl solutions with concentrations ranging from 0.01 mM to 3 M.** (a) COF-301/PAN, (b) $HB_{1.9}$@COF-301/PAN, (c) $HB_{3.8}$@COF-301/PAN, (d) $HB_{5.7}$@COF-301/PAN, and (e) $HB_{7.6}$@COF-301/PAN.

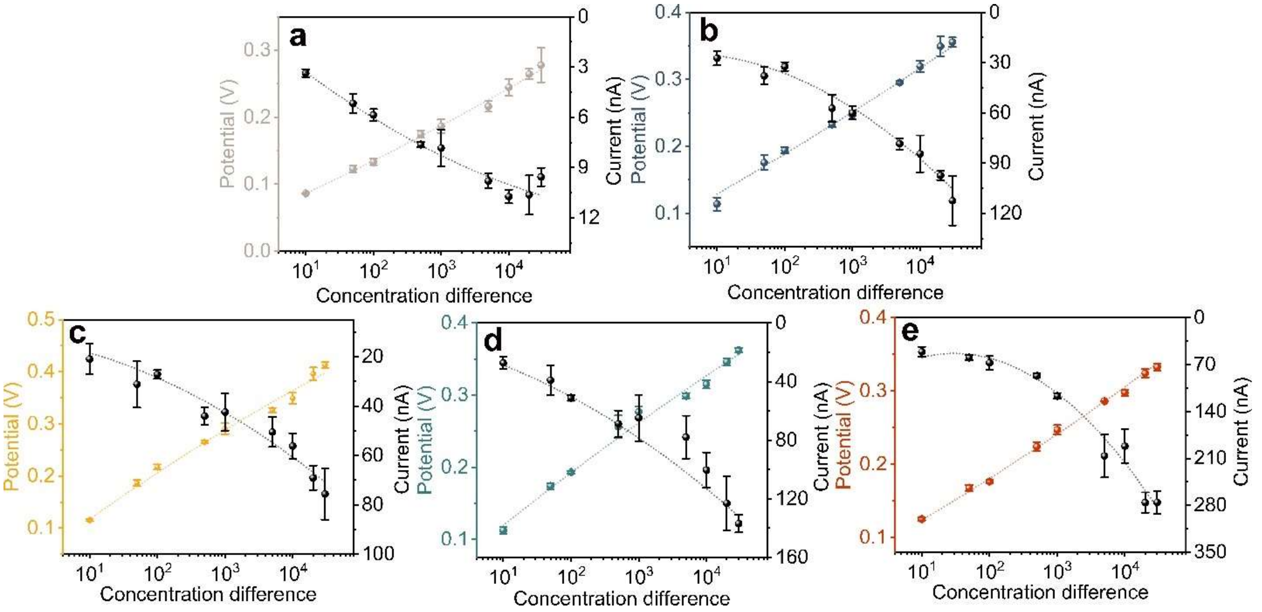


**Supplementary Figure 28| Plots of the recorded $V_{oc}$ and $I_{sc}$ versus KCl concentration difference.** (a) COF-301/PAN, (b) $HB_{1.9}$@COF-301/PAN, (c) $HB_{3.8}$@COF-301/PAN, (d) $HB_{5.7}$@COF-301/PAN, and (e) $HB_{7.6}$@COF-301/PAN. Error bars depict the standard deviation of three indi-vidual measurements

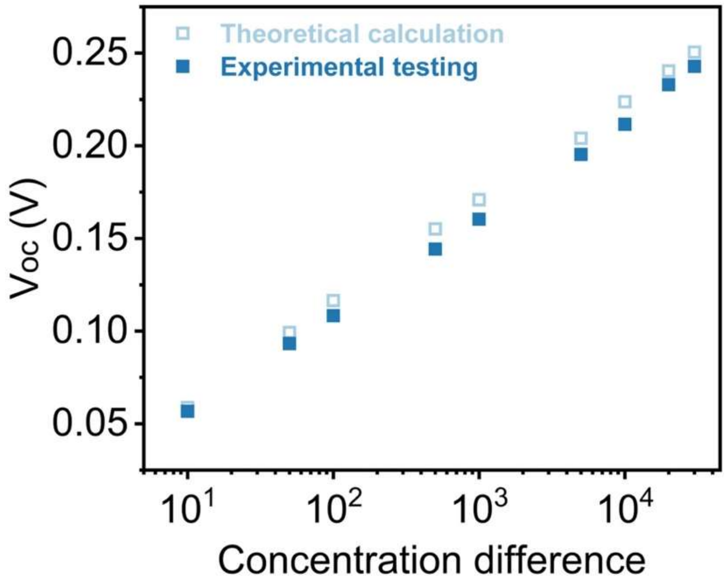


**Supplementary Figure 29 | The comparison of the experimental redox potential of Ag/AgCl over various concentration differences and the corresponding value calculated by the Nernst equation.**

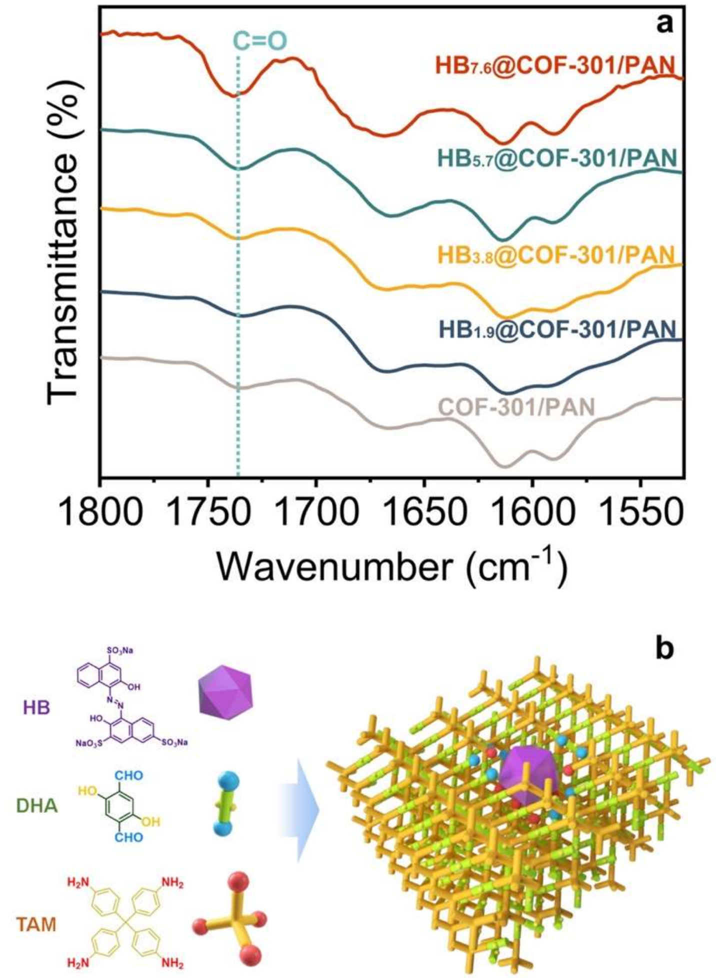


**Supplementary Figure 30 | Identification of the location of dye molecules.** (a) IR spectra of $HB_x$@COF-301/PAN and (b) the schemetic illustration of the poposed location of dye molecules in the membrane. The IR spectra show that the corresponding absorbance of the aldehyde C=O group (1735 $cm^{-1}$) in $HB_x$@COF-301/PAN became stronger as the HB content increased. This observation can be rationalized by considering the size of the dye molecule in comparison to the pore size of COF-301. Since the dye molecule is larger than the pore size of the COF-301 framework, it hinders the full condensation of the monomers. As a result, the presence of the dye leads to a higher concentration of unreacted aldehyde groups within the COF structure, which is reflected in the increased absorbance at 1735 $cm^{-1}$ in the IR spectra.

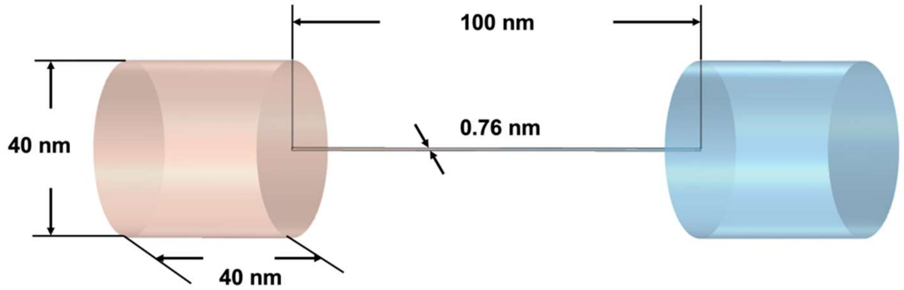


**Supplementary Figure 31 | Schematic of the 3D model used for numerical simulations.**

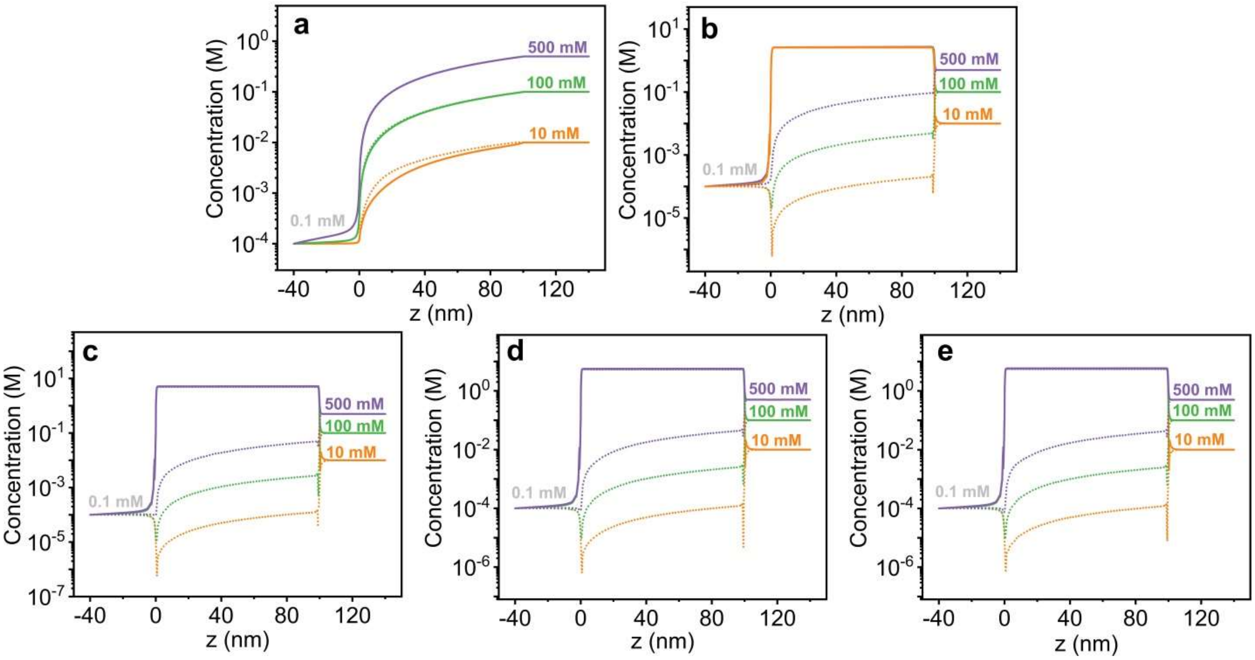


**Supplementary Figure 32 | Numerical simulation.** The distribution of $K^+$ (solid line) and $Cl^-$ (dashed line) at the center of the nanochannels of (a) COF-301/PAN, (b) $HB_{1.9}$@COF-301/PAN, (c) $HB_{3.8}$@COF-301/PAN, (d) $HB_{5.7}$@COF-301/PAN, and (e) $HB_{7.6}$@COF-301/PAN, respectively.

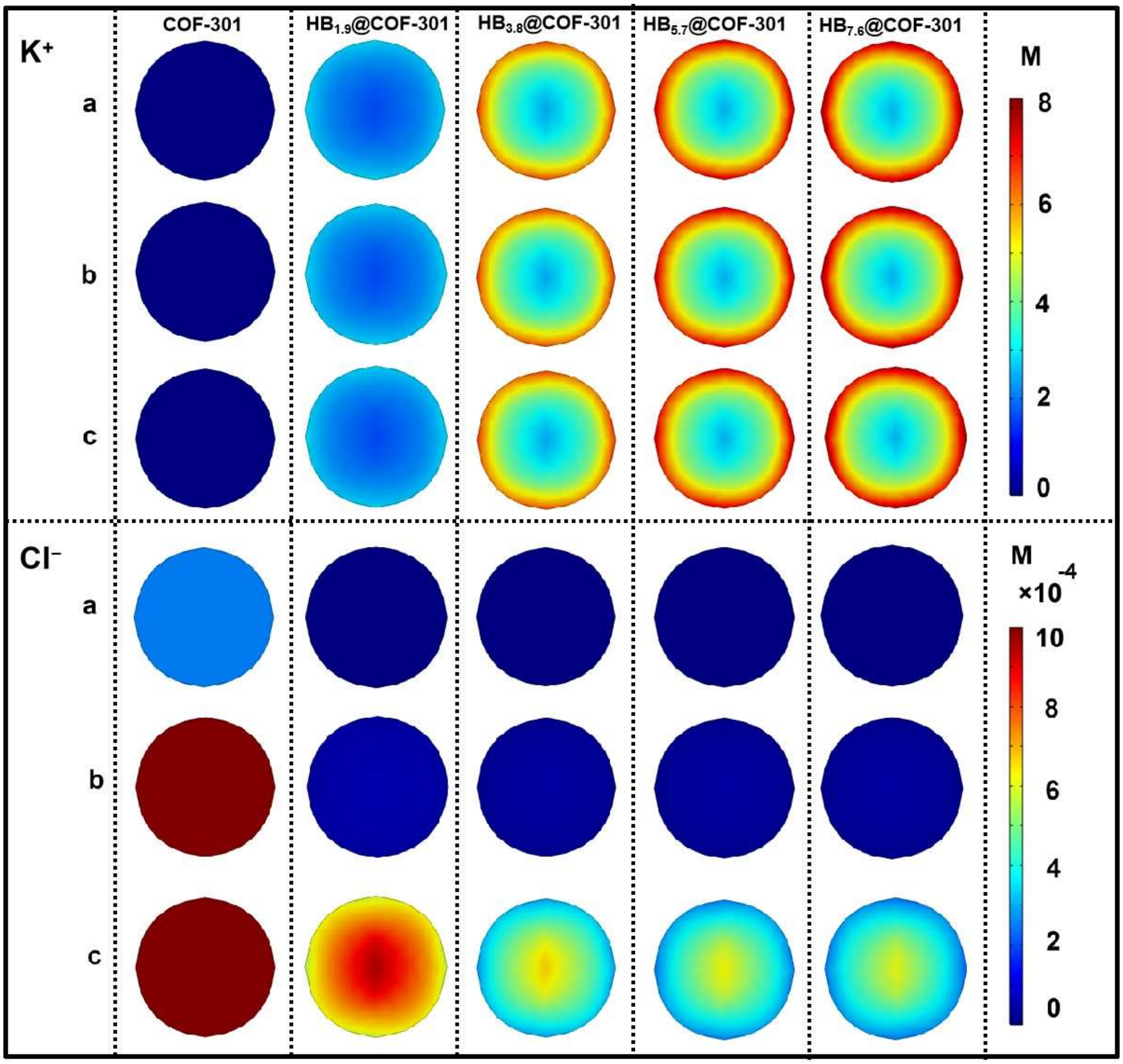


**Supplementary Figure 33 | Ion distribution.** The images of x–y cross-sectional concentration distributions of $K^+$ and $Cl^-$ at 0 nm along the z-axis (i.e. at the mouth of the low concentration side) over various concentration differences of (a) 0.1 mM/10 mM, (b) 0.1 mM/0.1 M, and (c) 0.1 mM/0.5 M, respectively.

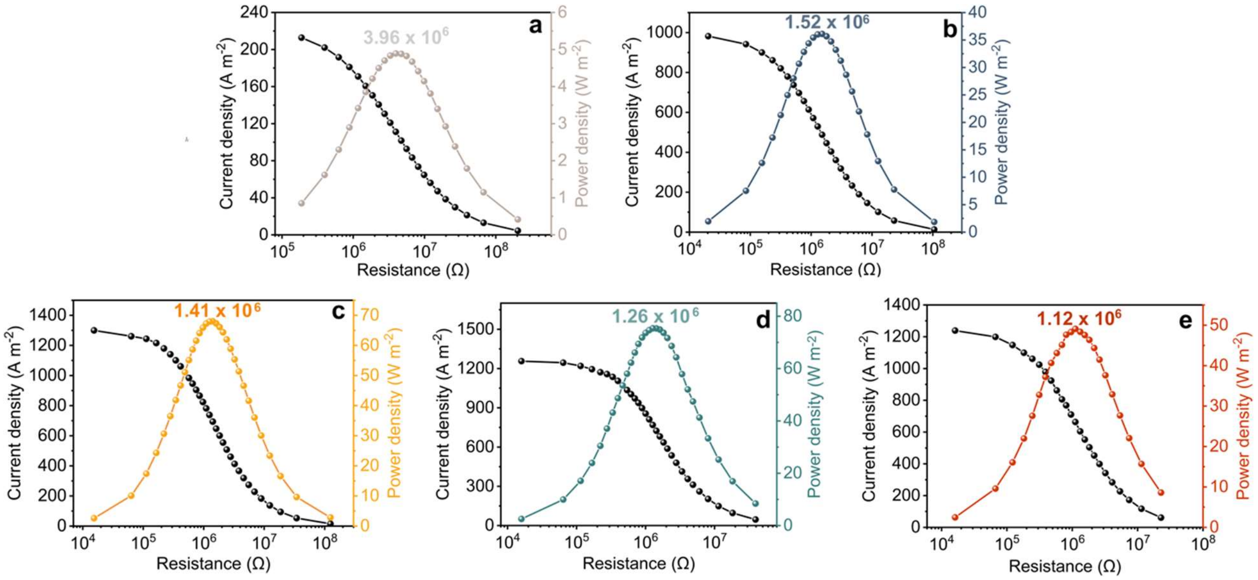


**Supplementary Figure 34 | The power was output to an external circuit to supply an electronic load.** The diffusion current of the nanofludic device decreased with increasing load resistance, and the output powers reached peaks at $R_L$ values of 3960, 1520, 1410, 1260, and 1120 kΩ for (a) COF-301/PAN, (b) $HB_{1.9}$@COF-301/PAN, (c) $HB_{3.8}$@COF-301/PAN, (d) $HB_{5.7}$@COF-301/PAN, and (e) $HB_{7.6}$@COF-301/PAN, respectively.

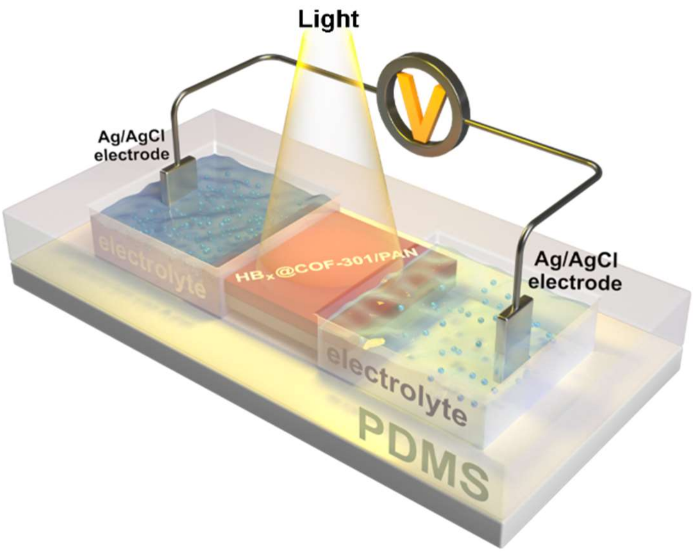


**Supplementary Figure 35 | Schematic of the experimental setup for measuring the transmembrane ionic transport under light illumination.** Light homogeneously irradiates the whole membrane.

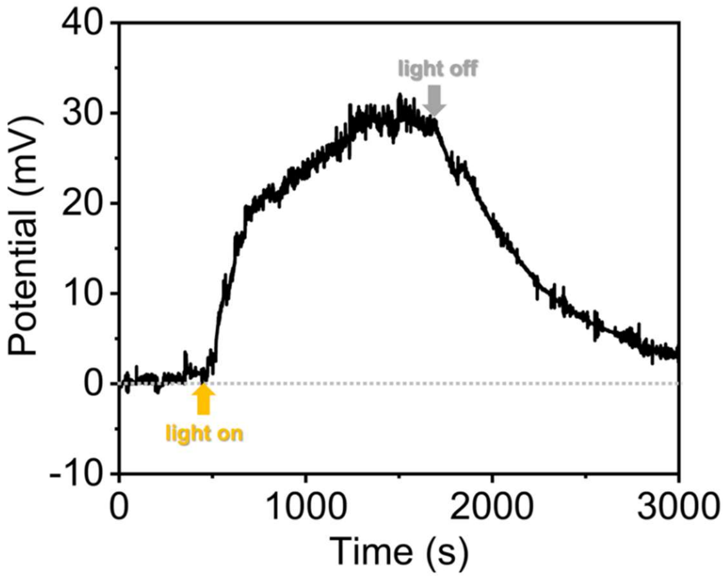


**Supplementary Figure 36 | $V$–$t$ curve.** The nanofluidic device assembled by $HB_{5.7}$@COF-301/PAN was measured at symmetric KCl aqueous solution (100 mmol) upon illumination with a 120 mW cm$^{-2}$ xenon lamp. The voltage increases when the light is turned on and drops when the light is switched off, reaching a value that is comparable with the "no light" reference experiment.

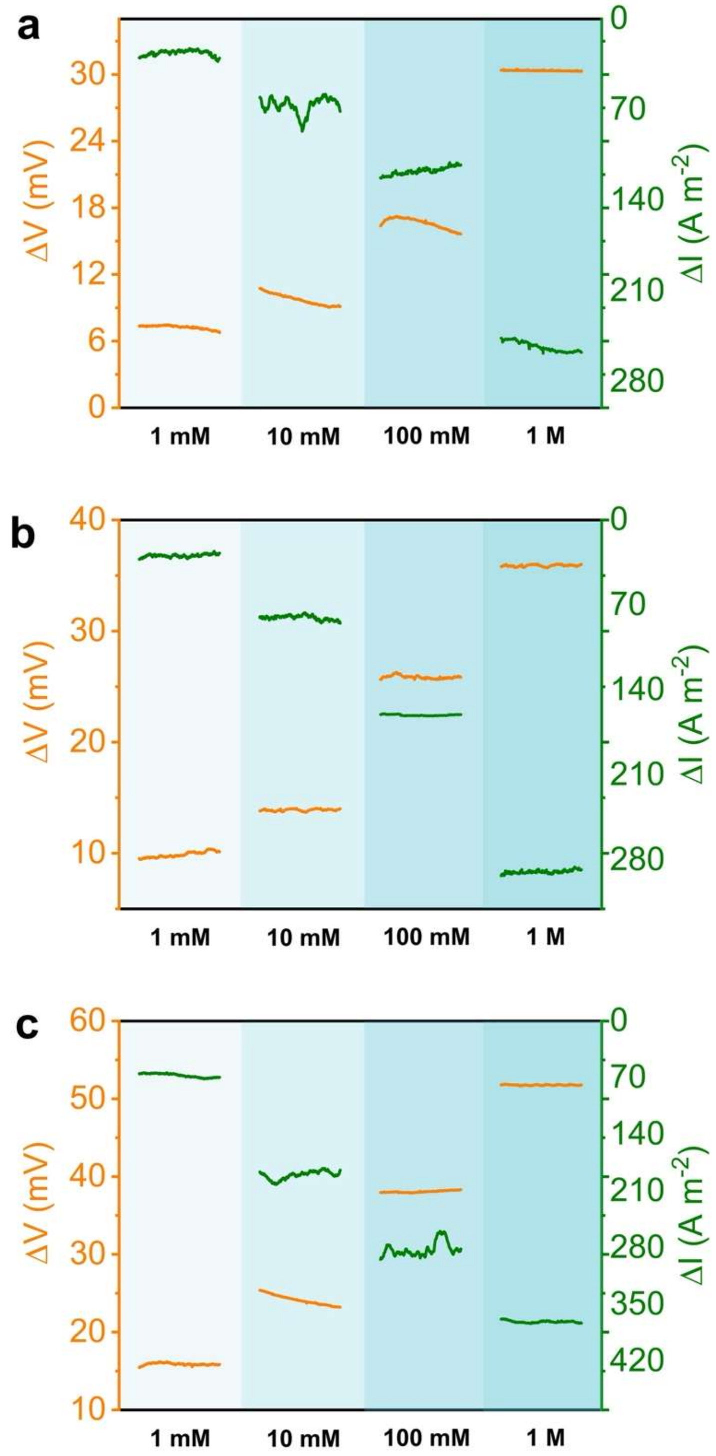


**Supplementary Figure 37 | The effect of light power on the photoresponsive behavior of the $HB_{5.7}$@COF-301/PAN membrane.** These selective regions depict the changes in voltage and current density measured in KCl aqueous solutions with equimolar concentrations ranging from 1 mM to 1 M (duration time: 200 s). The measurements were conducted under illumination with a xenon lamp at three different intensities: (a) 80 mW $cm^{-2}$, (b) 100 mW $cm^{-2}$, and (c) 150 mW $cm^{-2}$.

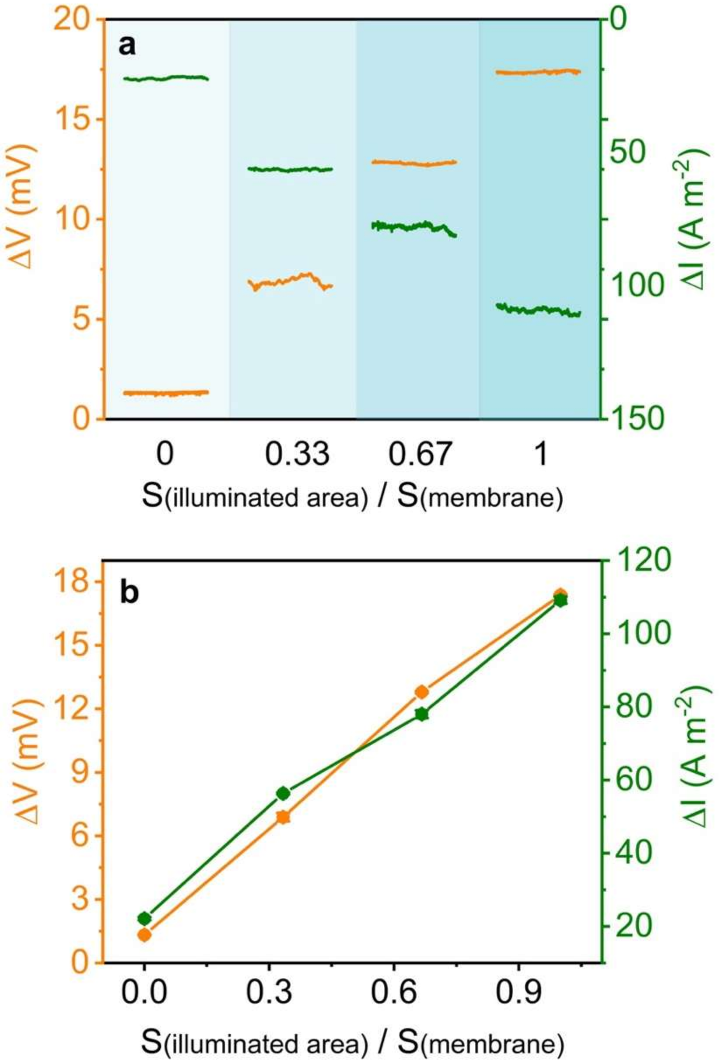


**Supplementary Figure 38 |** Exploring the impact of the illuminated membrane area on the light-induced photocurrent and photovoltage. (a) Selective region showcasing the changes in voltage and current density measured in 10 mM KCl aqueous solutions using the nanofluidic device constructed with $HB_{5.7}$@COF-301/PAN. The device was illuminated with a xenon lamp at an intensity of 120 mW $cm^{-2}$, while the illuminated membrane area varied. (b) Corresponding plots illustrating the average increase in voltage and current density as a function of the ratio of the illuminated membrane area to the total membrane area. Error bars depict the standard deviation of three indi-vidual measurements.

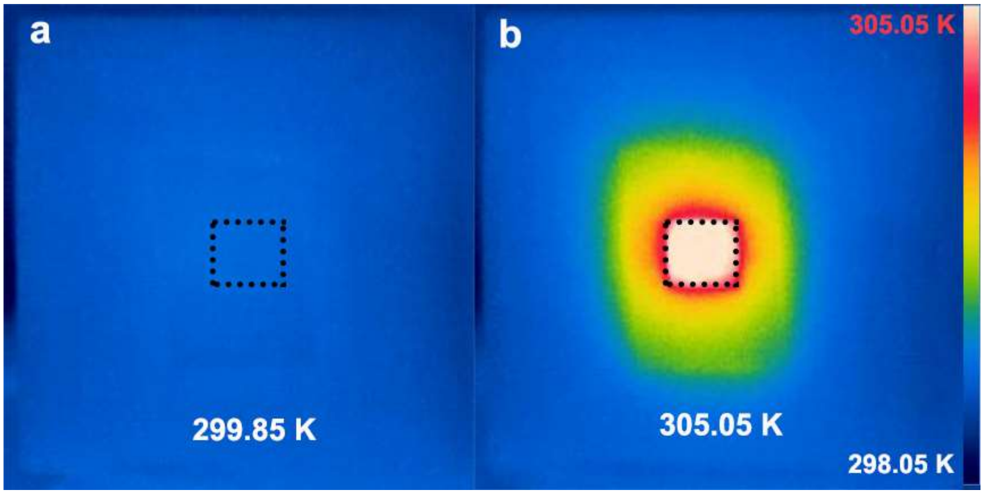


**Supplementary Figure 39 | Study of the photothermal effect on the membrane.** IR camera images (350 nm) illustrating the $HB_{5.7}$@COF-301/PAN membrane before (a) and after (b) exposure to illumination from a xenon lamp with an intensity of 120 mW $cm^{-2}$. The images capture the gradual rise in membrane temperature throughout the irradiation process, reaching a stable state after 5 minutes with a maximum temperature increase of 5.2 K.

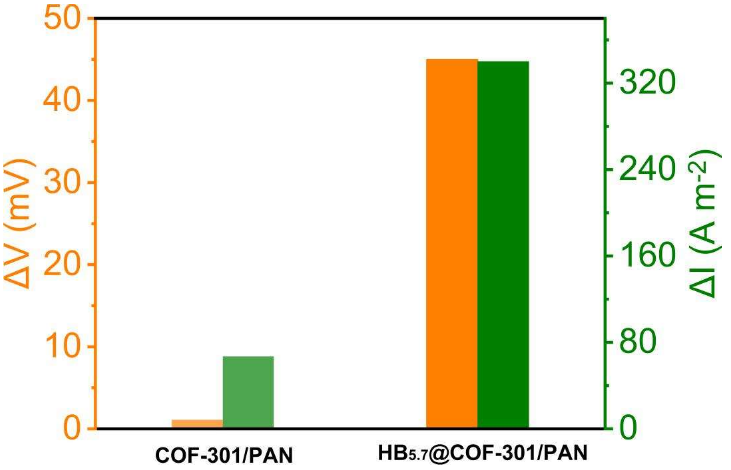


**Supplementary Figure 40 | The comparison of photogating phenomena of various materials.** The maximum increments of voltage and current density of the nanofluidic device assembled by COF-301/PAN and $HB_{5.7}$@COF-301/PAN upon illumination with a 120 mW $cm^{-2}$ xenon lamp measured at symmetric KCl aqueous solution (1 M).

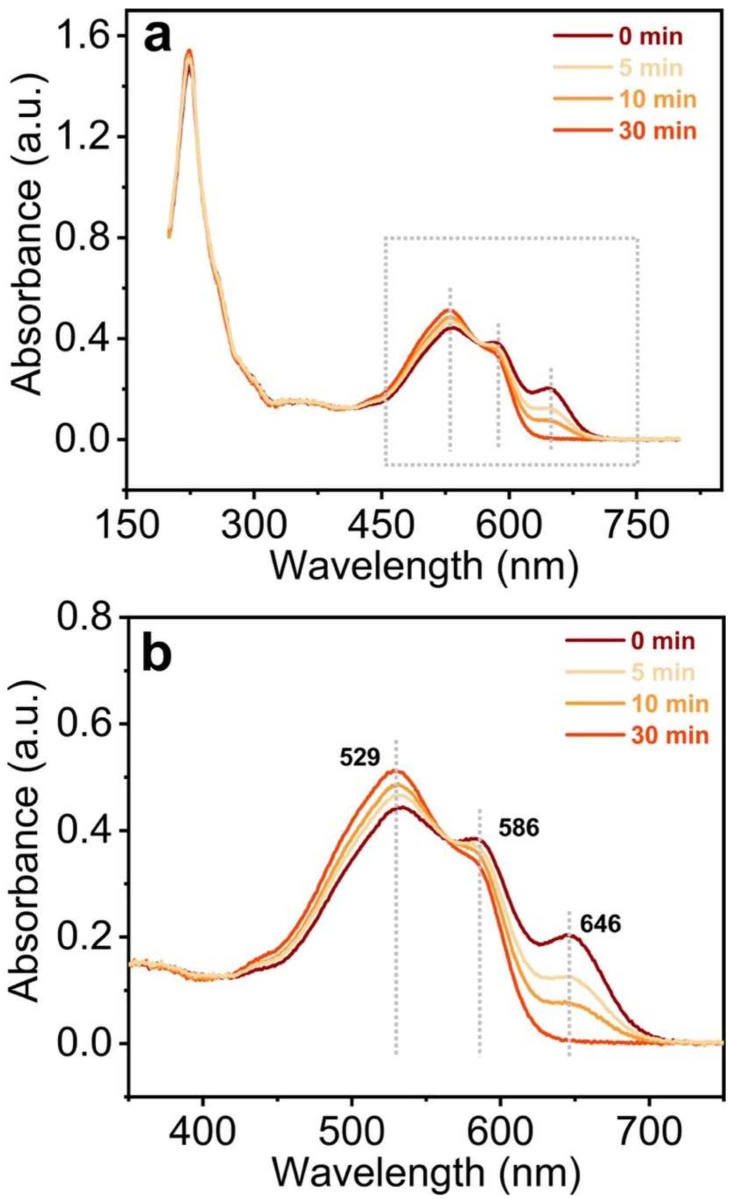


**Supplementary Figure 41 | UV-vis spectra.** (a) The evolution of UV-vis spectra of HB aqueous solution under light illumination and (b) enlarged section of gray rectangle in (a).

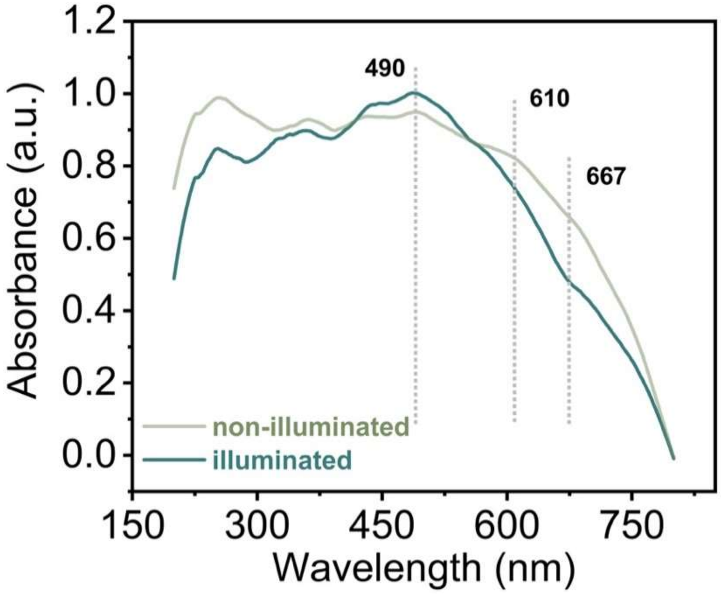


**Supplementary Figure 42 | UV-vis spectra of the $HB_{5.7}$@COF-301/PAN membrane before and after illumination with a 120 mW cm$^{-2}$ xenon lamp for 30 min.** We observed a rapid decrease in the intensity of peaks at 610 and 667 nm, accompanied by an increase in the peak at 490 nm. Although the peak positions of HB in the liquid UV-vis spectra changed to 586, 646, and 529 nm, the overall trend of the changes remained consistent. We attribute these peak position variations to the influence of host-guest interactions between the HB molecules and the COF-301 host, as well as the presence of $H_2O$ in the liquid UV-vis spectroscopy. It is worth noting that different solvents can induce shifts in the absorption maxima or even lead to the formation of distinct species.

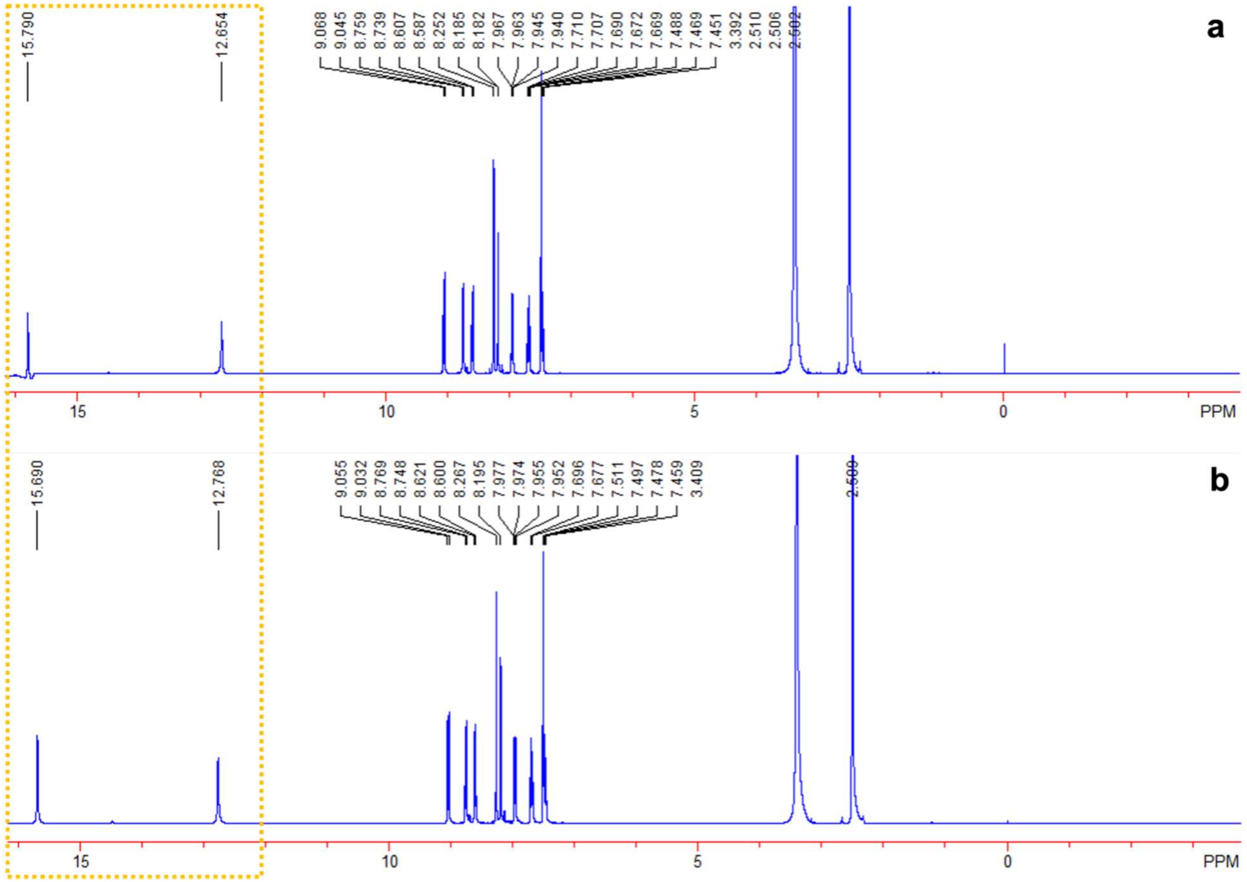


**Supplementary Figure 43 | $^1$H NMR spectra of HB.** (a) Before and (b) after being illuminated under a 120 mW cm$^{-2}$ xenon lamp for 30 min. Orange rectangle: the chemical shifts of the OH resonances.

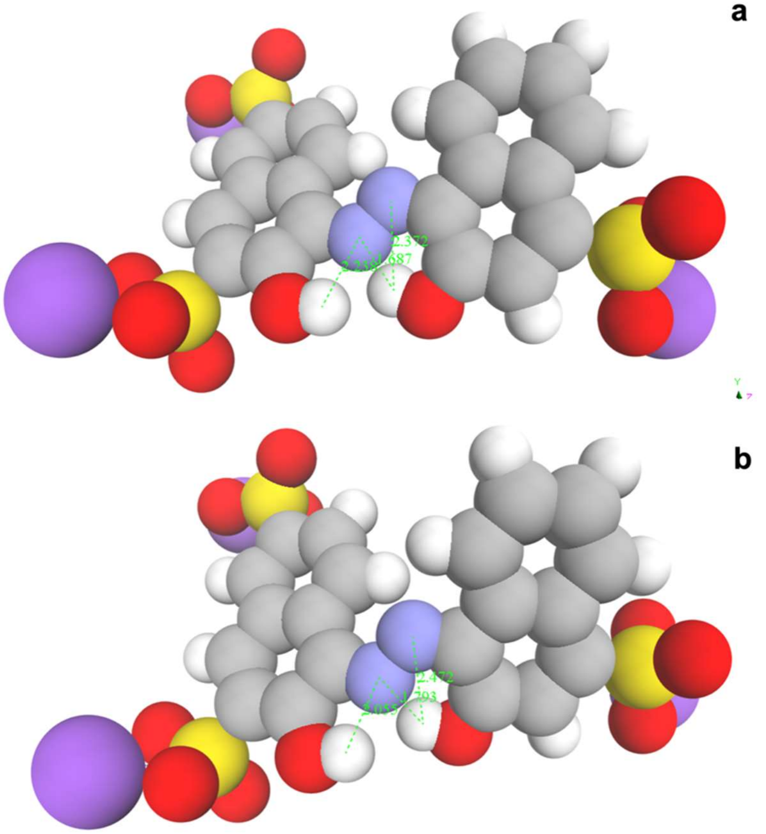


**Supplementary Figure 44 | DFT simulation.** The optimized HB molecule structure of (a) ground state and (b) excited state (blue, N; grey, C; red, O; white, H; yellow, S; purple, Na). Green line: the lengths of H-bonds between the phenolic hydroxyl groups and the azo moiety. The calculations were optimized at cam-b3lyp/6-31+g(d,2p) level in the Gaussian 16 program [Frisch, M.J., Trucks, G.W., Schlegel, H.B., Scuseria, G.E., Robb, M.A., Cheeseman, J.R., Scalmani, G., Barone, V., Petersson, G.A., Nakatsuji, H., et al. (2016). Gaussian (Inc. Wallingford CT)].

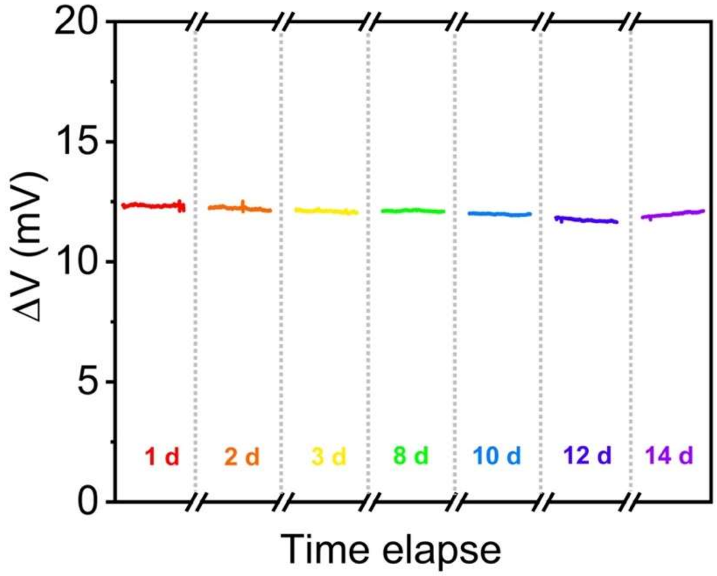


**Supplementary Figure 45 | Stability evaluation.** Time series plots of voltage increment versus time ($V–t$) for the nanofluidic device assembled by $HB_{5.7}$@COF-301/PAN under an identical KCl concentration of 1 mmol upon illumination with a 120 mW $cm^{-2}$ xenon lamp.

## ***Supplementary references***


1. Cao, L. et al. Giant osmotic energy conversion through vertical-aligned ion-permselective nanochannels in covalent organic framework membranes. *J. Am. Chem. Soc.* **144**, 12400–12409 (2022).
2. Cao, L. et al. An ionic diode covalent organic framework membrane for efficient osmotic energy conversion. *ACS Nano* **11**, 18910–18920 (2022).
3. Ding, L. et al. Oppositely charged $Ti_3C_2T_x$ MXene membranes with 2D nanofluidic channels for osmotic energy harvesting. *Angew. Chem. Int. Ed.* **61**, e202206152 (2022).
4. Zhang, Z. et al. Cation-selective two-dimensional polyimine membranes for high-performance osmotic energy conversion. *Nat Commun.* **13**, 3935 (2022).
5. Xie, L. et al. Sequential superassembly of nanofiber arrays to carbonaceous ordered mesoporous nanowires and their heterostructure membranes for osmotic energy conversion. *J. Am. Chem. Soc.* **143**, 6922–6932 (2021).
6. Wang, Z. et al. On-water surface synthesis of charged two-dimensional polymer single crystals via the irreversible Katritzky reaction. *Nat. Synth.* **1**, 69–76 (2022).
7. Sun, Y. et al. Tailoring a poly(ether sulfone) bipolar membrane: osmotic-energy generator with high power density. *Angew. Chem. Int. Ed.* **59**, 17423–17428 (2020).
8. Chen, C. et al. Bio-inspired nanocomposite membranes for osmotic energy harvesting. *Joule* **4**, 247–261 (2020).
9. Zhang, Z. et al. Oxidation promoted osmotic energy conversion in black phosphorus membranes. *Proc. Natl. Acad. Sci. U.S.A.* **117**, 13959–13966 (2020).
10. Feng, J. et al. Single-layer $MoS_2$ nanopores as nanopower generators. *Nature* **536**, 197–200 (2016).
11. Yang, J. et al. Advancing osmotic power generation by covalent organic framework monolayer. *Nat. Nanotechnol.* **17**, 622–628 (2022).
12. Chen, S. et al. Imparting ion selectivity to covalent organic framework membranes using de novo assembly for blue energy harvesting. *J. Am. Chem. Soc.* **25**, 9415–9422 (2021).
13. Liu, P. et al. Neutralization reaction assisted chemical-potential-driven ion transport through layered titanium carbides membrane for energy harvesting. *Nano Lett.* **20**, 3593–3601 (2020).
14. Palenzuela, P. et al. Performance analysis of a red-med salinity gradient heat engine. *Energies* **11**, 3385 (2018).
15. Yang, G. et al. Stable $Ti_3C_2T_x$ MXene–boron nitride membranes with low internal resistance for enhanced salinity gradient energy harvesting. *ACS Nano* **15**, 6594–6603 (2021).
16. Xin, W. et al. Biomimetic nacre-like silk-crosslinked membranes for osmotic energy harvesting. *ACS Nano* **14**, 9701–9710 (2020).
17. Van Toan, N. et al. Thermoelectric power battery using $Al_2O_3$ nanochannels of 10 nm diameter for energy harvesting of low-grade waste heat. *Energy Convers. Manag.* **199**, 111979 (2019).

18. Li, Z.-Q. et al. A solar thermoelectric nanofluidic device for solar thermal energy harvesting. *CCS Chem*. **2**, 2174–2182 (2020).
19. Chen, K. et al. Thermo-osmotic energy conversion and storage by nanochannels. *J. Mater. Chem. A* **7**, 25258–25261 (2019).
20. Wu, C. et al. Biomimetic temperature-gated 2D cationic nanochannels for controllable osmotic power harvesting. *Nano Energy* **6**, 105113 (2020).
21. Mai, V.-P. & Yang, R.-J. Boosting power generation from salinity gradient on high-density nanoporous membrane using thermal effect. *Applied Energy* **274**, 115294 (2020).
22. Long, R., Luo, Z., Kuang, Z., Liu, Z. & Liu, W. Effects of heat transfer and the membrane thermal conductivity on the thermally nanofluidic salinity gradient energy conversion. *Nano Energy* **67**, 104284 (2020).
23. Sun, Y. et al. Thermoenhanced osmotic power generator via lithium bromide and asymmetric sulfonated poly(ether ether ketone)/poly(ether sulfone) nanofluidic membrane. *NPG Asia Mater.* **13**, 50 (2021).
24. Zuo, X. et al. Thermo-osmotic energy conversion enabled by covalent-organic-framework membrane with record output power density. *Angew. Chem. Int. Ed.* **61**, e202116910 (2022)
25. Liu, P. et al. Synergy of light and acid-base reaction in energy conversion based on cellulose nanofiber intercalated titanium carbide composite nanofluidics. *Energy Environ. Sci.* **14**, 4400–4409 (2021).
26. Xie, X., Crespo, G. A., Mistlberger, G. & Bakker, E. Photocurrent generation based on a light-driven proton pump in an artificial liquid membrane. *Nat. Chem.* **6**, 202–207 (2014).
27. Xiao, K. et al. Artificial light-driven ion pump for photoelectric energy conversion. *Nat Commun.* **10**, 74 (2019).
28. Li, Z.-Q. et al. Light-enhanced osmotic energy harvester using photoactive porphyrin metal–organic framework membranes. *Angew. Chem. Int. Ed.* **61**, e202202698 (2022).
29. Liu, P. et al. Light-induced heat driving active ion transport based on 2D MXene nanofluids for enhancing osmotic energy conversion. *CCS Chem.* **2**, 1325–1335 (2020).
30. Graf, M. et al. Light-enhanced blue energy generation using $MoS_2$ nanopores. *Joule* **3**, 1549–1564 (2019).
31. Zhang, D., Ren, Y., Fan, X., Zhai, J. & Jiang, L. Photoassisted salt-concentration-biased electricity generation using cation-selective porphyrin-based nanochannels membrane. *Nano Energy* **76**, 105086 (2020).
32. Xiao, K. et al. Light-driven directional ion transport for enhanced osmotic energy harvesting. *Natl. Sci. Rev.* **8**, nwaa231 (2020).
